# Senolytics reduce coronavirus-related mortality in old mice

**DOI:** 10.1126/science.abe4832

**Published:** 2021-06-08

**Authors:** Christina D. Camell, Matthew J. Yousefzadeh, Yi Zhu, Larissa G. P. Langhi Prata, Matthew A. Huggins, Mark Pierson, Lei Zhang, Ryan D. O’Kelly, Tamar Pirtskhalava, Pengcheng Xun, Keisuke Ejima, Ailing Xue, Utkarsh Tripathi, Jair Machado Espindola-Netto, Nino Giorgadze, Elizabeth J. Atkinson, Christina L. Inman, Kurt O. Johnson, Stephanie H. Cholensky, Timothy W. Carlson, Nathan K. LeBrasseur, Sundeep Khosla, M. Gerard O’Sullivan, David B. Allison, Stephen C. Jameson, Alexander Meves, Ming Li, Y. S. Prakash, Sergio E. Chiarella, Sara E. Hamilton, Tamara Tchkonia, Laura J. Niedernhofer, James L. Kirkland, Paul D. Robbins

**Affiliations:** 1 Institute on the Biology of Aging and Metabolism, Department of Biochemistry, Molecular Biology and Biophysics, University of Minnesota, Minneapolis, MN, USA.; 2 Robert and Arlene Kogod Center on Aging, Mayo Clinic, Rochester, MN, USA.; 3 Department of Physiology and Biomedical Engineering, Mayo Clinic, Rochester, MN, USA.; 4 Department of Laboratory Medicine and Pathology and Center of Immunology, University of Minnesota, Minneapolis, MN, USA.; 5 Department of Epidemiology and Biostatistics, School of Public Health, Indiana University–Bloomington, Bloomington, IN, USA.; 6 Division of Biomedical Statistics and Informatics, Department of Health Sciences Research, Mayo Clinic, Rochester, MN, USA.; 7 Masonic Cancer Center Comparative Pathology Shared Resource, University of Minnesota, St. Paul, MN, USA.; 8 Department of Veterinary Population Medicine, University of Minnesota, St. Paul, MN, USA.; 9 Department of Physical Medicine and Rehabilitation, Mayo Clinic, Rochester, MN, USA.; 10 Division of Endocrinology, Department of Medicine, Mayo Clinic, Rochester, MN, USA.; 11 Department of Dermatology, Mayo Clinic, Rochester, MN, USA.; 12 Department of Anesthesiology and Perioperative Medicine, Mayo Clinic, Rochester, MN, USA.; 13 Division of Allergic Diseases, Department of Medicine, Mayo Clinic, Rochester, MN, USA.; 14 Division of General Internal Medicine, Department of Medicine, Mayo Clinic, Rochester, MN, USA.

## Abstract

The COVID-19 pandemic has revealed the pronounced vulnerability of the elderly and chronically ill to severe acute respiratory syndrome coronavirus 2 (SARS-CoV-2)–induced morbidity and mortality. Cellular senescence contributes to inflammation, multiple chronic diseases, and age-related dysfunction, but effects on responses to viral infection are unclear. Here, we demonstrate that senescent cells (SnCs) become hyper-inflammatory in response to pathogen-associated molecular patterns (PAMPs), including SARS-CoV-2 spike protein-1, increasing expression of viral entry proteins and reducing antiviral gene expression in non-SnCs through a paracrine mechanism. Old mice acutely infected with pathogens that included a SARS-CoV-2–related mouse β-coronavirus experienced increased senescence and inflammation, with nearly 100% mortality. Targeting SnCs by using senolytic drugs before or after pathogen exposure significantly reduced mortality, cellular senescence, and inflammatory markers and increased antiviral antibodies. Thus, reducing the SnC burden in diseased or aged individuals should enhance resilience and reduce mortality after viral infection, including that of SARS-CoV-2.

## Senescent cells exacerbate COVID-19

Cellular senescence is a state elicited in response to stress signals and is associated with a damaging secretory phenotype. The number of senescent cells increases with advanced age and this in turn drives age-related diseases. Camell *et al.* show that senescent cells have an amplified inflammatory response to severe acute respiratory syndrome coronavirus 2 (SARS-CoV-2) (see the Perspective by Cox and Lord). This response is communicated to nonsenescent cells, suppressing viral defense mechanisms and increasing the expression of viral entry proteins. In old mice infected with a SARS-CoV-2–related virus, treatment with senolytics to reduce the senolytic cell burden reduced mortality and increased antiviral antibodies.

*Science*, abe4832, this issue p. eabe4832; see also abi4474, p. 281

## Abstract (structured)

**INTRODUCTION:**

The COVID-19 pandemic revealed enhanced vulnerability of the elderly and chronically ill to adverse outcomes upon severe acute respiratory syndrome coronavirus 2 (SARS-CoV-2) infection. Senescence is a cell fate elicited by cellular stress that results in changes in gene expression, morphology, metabolism, and resistance to apoptosis. Senescent cells (SnCs) secrete pro-inflammatory factors, called the senescence-associated secretory phenotype (SASP). SnCs accumulate with age and drive chronic inflammation. In human cells and tissues and using a new infection paradigm, we asked whether SnCs are a cause of adverse outcomes of infection with aging. This is relevant because SnCs can be selectively eliminated in vivo with a new class of therapeutics called senolytics, potentially affording a new approach to treat COVID-19.

**RATIONALE:**

We hypothesized that SnCs, because of their pro-inflammatory SASP, might have a heightened response to pathogen-associated molecular pattern (PAMP) factors, resulting in increased risk of cytokine storm and multi-organ failure. To test this, we treated senescent and nonsenescent human cells with the PAMPs lipopolysaccharide (LPS) and SARS-CoV-2 spike protein (S1) and measured the SASP and its effect on non-SnCs. Similarly, old and progeroid mice were challenged with LPS, and we measured the SASP. Previously, we created a “normal microbial experience” (NME) for mice by transmitting environmental pathogens to specified-pathogen–free (SPF) mice through exposure to pet store mice or their bedding. The first pathogen transferred was mouse hepatitis virus (MHV), a β-coronavirus closely related to SARS-CoV-2. NME rapidly killed aged SPF mice known to have an increased burden of SnCs compared with young SPF mice, which survive NME. This afforded an experimental paradigm to test whether senolytics blunt adverse outcomes in β-coronavirus infection.

**RESULTS:**

Human endothelial SnCs became hyperinflammatory in response to challenge with LPS and S1, relative to non-SnCs. The PAMP-elicited secretome of SnCs caused increased expression of viral entry proteins and reduced expression of antiviral genes in nonsenescent human endothelial and lung epithelial cells, and the proximity of these events was established in human lung biopsies. Treatment of old mice with LPS significantly increased SASP expression in several organs relative to young mice, confirming our hypothesis in vivo. Similarly, old mice exposed to NME displayed a significant multi-organ increase in SnCs and the SASP, impaired immune response to MHV, and 100% mortality, whereas inoculation with antibodies against MHV before NME afforded complete rescue of mortality. Treating old mice with the senolytic fisetin, which selectively eliminates SnCs after NME reduced mortality by 50%, reduced expression of inflammatory proteins in serum and tissue and improved the immune response. This was confirmed with a second senolytic regimen, Dasatinib plus Quercetin, as well as genetic ablation of SnCs in aged mice, establishing SnCs as a cause of adverse outcomes in aged organisms exposed to a new viral pathogen.

**CONCLUSION:**

SnCs amplify susceptibility to COVID-19 and pathogen-induced hyperinflammation. Reducing SnC burden in aged mice reduces mortality after pathogen exposure, including a β-coronavirus. Our findings strongly support the Geroscience hypothesis that therapeutically targeting fundamental aging mechanisms improves resilience in the elderly, with alleviation of morbidity and mortality due to pathogenic stress. This suggests that senolytics might protect others vulnerable to adverse COVID-19 outcomes in whom increased SnCs occur (such as in obesity or numerous chronic diseases).

## Teaser

SARS-CoV-2 amplifies the damaging senescent cell secretory state, and drugs that selectively clear senescent cells reduce mortality in infected aged mice.

## Graphical abstract

**Figure:**
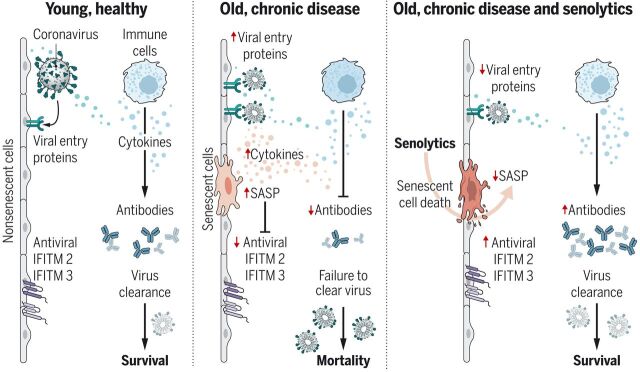
SnCs that accumulate with age or chronic disease react to PAMPs such as SARS-CoV-2 S1 by amplifying the SASP, which increases viral entry protein expression and decreases viral defense IFITMs in normal cells. Old mice exposed to pathogens such as the β-coronavirus MHV have increased inflammation and higher mortality. Treatment with a senolytic decreased SnCs, inflammation, and mortality and increased the antiviral antibody response.

Old age is the greatest risk factor by orders of magnitude for most chronic diseases, including cancers, diabetes, cardiovascular disease, and Alzheimer’s disease. Aging also predisposes to geriatric syndromes and loss of physical resilience. The current COVID-19 pandemic has illuminated the particular vulnerability of the elderly and those with underlying geriatric syndromes to increased severe acute respiratory syndrome coronavirus 2 (SARS-CoV-2)–mediated mortality (*1*–*5*). Thus, approaches to extend health span and enhance physical resilience could reduce the rate of mortality in elderly COVID-19 patients.

Cellular senescence has emerged as one of the mechanisms that drives aging and age-related diseases that is most tractable to therapeutically target (*6*, *7*). Senescence is a cell fate elicited in response to external and internal cellular stress signals, established through transcription factor cascades that can include p16^INK4a^/retinoblastoma protein and/or p53/p21^CIP1^, which cause extensive changes in gene expression, histone modifications, organelle function, elevated protein production, and profound morphologic and metabolic shifts (*8*, *9*). A substantial fraction of senescent cells (SnCs) release inflammatory factors, chemokines, growth factors, proteases, bioactive lipids, extracellular vesicles, and procoagulant factors, called the senescence-associated secretory phenotype (SASP) (*6*).

Senescence is a robust tumor suppressor mechanism, with the SASP acting as a chemoattractant-stimulating immune cell–mediated clearance of senescent and neighboring cells. However, with advancing age and many chronic diseases, SnCs accumulate in most tissues, presumably because of inefficient SnC removal by the immune system and resistance to cell death. This accumulation drives chronic sterile inflammation, which in turn drives loss of resilience and predisposition to many diseases (*10*). SnCs can interfere with the immune system and the ability of immune cells to remove them. For example, the SASP factors interleukin-6 (IL-6), monocyte chemotactic protein–1 (MCP-1), and chemokine (C-C motif) ligand 11 (CCL11) alter myeloid cell migration; interferon γ-induced protein 10 (IP10)/C-X-C motif chemokine 10 (CXCL10) depletes critical T lymphocyte subsets; and matrix metalloproteinases cleave fatty acid synthase (FAS) ligand and other immune system regulators (*11*). The SASP can drive fibrosis (*11*). SnCs have been demonstrated to play a causal role in aging and age-related diseases in preclinical models. Transplanting SnCs into young mice causes an accelerated aging-like state, whereas genetic or pharmacologic selective killing of SnCs attenuates disease, improves physical function, and delays all-cause mortality in older mice (*12*–*14*). Factors that are common components of the SASP are linked to prolonged disease, hyperinflammation/cytokine storm/acute respiratory distress syndrome (ARDS), myocarditis with troponin leak, T cell deficiencies, clotting, delirium, and multi-organ failure in SARS-CoV-2 patients (*15*). Also, a signature of the SASP factors IL-6, IL-10, and IP10 in COVID-19 patients appears to predict clinical progression (*16*). However, it is not known whether SnCs and their pro-inflammatory SASP contribute to the increased mortality observed in the elderly and chronically diseased after infection.

Initially, to determine whether SnCs have an altered response to pathogen exposure compared with healthy cells, we treated irradiation-induced senescent human preadipocytes and non-SnCs with the pathogen-associated molecular pattern (PAMP) factor lipopolysaccharide (LPS). LPS stimulated expression of *IL1*α, *IL1*β, *IL6*, *MCP1*, and *PAI2* in non-SnCs (Fig. 1A, fig. S1, and table S1) but did not significantly alter levels of *p16^INK4a^* or *p21^CIP1^*. Expression of these SASP factors as well as *IL10* and *PAI1* were all significantly increased by LPS in SnCs relative to untreated SnCs and relative to LPS-treated non-SnCs, suggesting that PAMPs exacerbate the SASP and that SnCs can amplify the inflammatory response to PAMPs. To determine whether a similar effect occurs in vivo, young and aged wild-type (WT) mice were challenged with LPS. Senescence and SASP markers were measured 24 hours after treatment. Although expression of the senescence markers *p16^Ink4a^* and *p21^Cip1^* was not affected at this early time point, LPS exposure stimulated a significant increase in expression of *Il1*α, *Il1*β, *Il6*, *Il10*, *Mcp1*, *Tnf*α, *Pai1*, and *Pai2* in liver (Fig. 1B) and kidney (fig. S2) of aged compared with young mice. Furthermore, LPS challenge significantly increased levels of the SASP factors IL-6, MCP-1, and tumor necrosis factor–α (TNFα) in the serum of old mice (Fig. 1C). To confirm the effect of LPS on aged mice with an increased SnC burden, we also treated progeroid *Ercc1^–/^*^∆^ mice (fig. S3)—which express high levels of senescence and SASP markers in the same tissues and to the same extent as occurs in WT mice, albeit much earlier in life (*17*)—acutely with LPS. Senescence and SASP markers were measured 24 hours after treatment. Although expression of senescence markers *p16^Ink4a^* and *p21^Cip1^* was not affected at this early time point, LPS significantly increased expression of SASP factors (*Il1*α, *Il1*β, *Il6*, *Tnf*α, and *Mcp1*) in kidney and liver of the progeroid mice relative to age-matched WT controls (fig. S3) and significantly increased levels of circulating IL-6 and MCP-1 (fig. S3). On the basis of these results, we hypothesized that SnCs exposed to pathogen-associated signals contribute to hyperinflammation and cytokine storm after infection with pathogens.

**Fig. 1:**
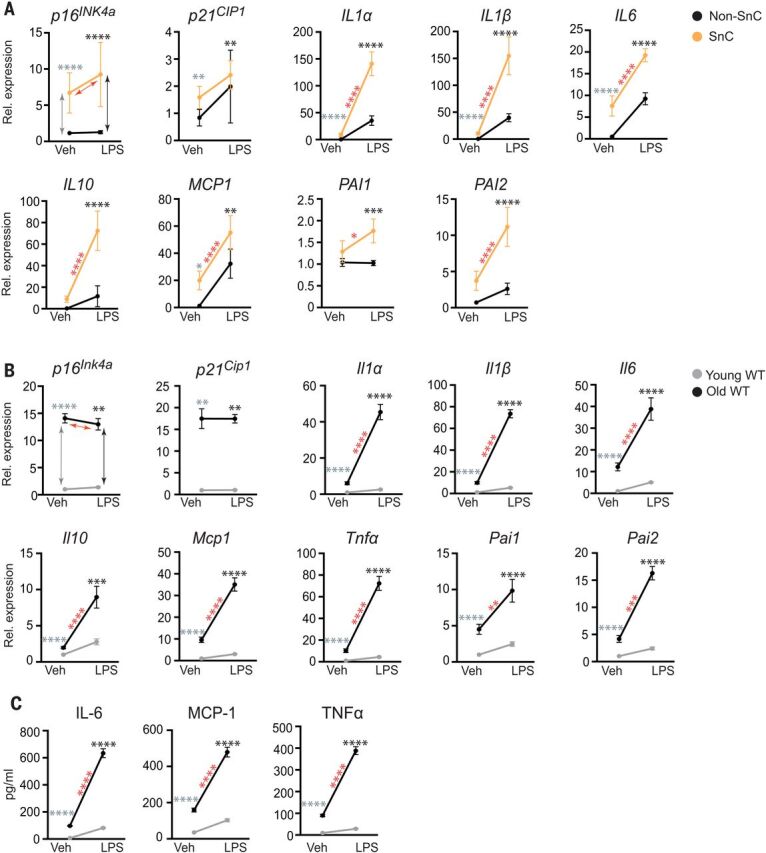
The SASP is amplified by PAMP factors. (**A**) Human adipocyte progenitors isolated from subcutaneous fat biopsies were induced to undergo senescence with 10 gray (Gy) of ionizing radiation (SnC) or not (non-SnC) (*n* = 5 subjects). Cells were treated with 10 ng of the prototype PAMP LPS for 3 hours before RNA isolation. Gene expression was measured with quantitative PCR, and the expression in LPS-treated cells was normalized to vehicle-treated samples. Means ± SEM. Statistical significance was calculated by using a mixed effect model for the effect of LPS on SnCs and its differential effects on SnCs compared with non-SnCs. Details are available in table S1. Arrows and asterisks: gray, vehicle-treated SnCs versus non-SnCs; black, LPS-treated SnCs versus non-SnCs; red, SnCs ± LPS. *P < 0.05, **P < 0.01, ***P < 0.001, ****P < 0.0001. (**B**) Young (2-month-old) and old (26-month-old) mice were treated with phosphate-buffered saline (PBS) (*n* = 5 young and 5 old) or LPS (*n* = 4 young and 3 old), and tissues were collected 24 hours later. RNA was isolated from liver, and gene expression measured by means of quamtitative PCR. Expression in LPS-treated mice was normalized to vehicle-treated animals. Means ± SEM, two-way analysis of variance (ANOVA) and post hoc comparison Tukey’s honestly significant difference used to compare the two animal cohorts within a treatment group. Arrows and asterisks: gray, vehicle-treated old versus young; black, LPS-treated old versus young; red, old ± LPS. **P < 0.01, ***P < 0.001, ****P < 0.0001. Kidney data are provided in fig. S2. (**C**) Serum protein from the same mice measured with enzyme-linked immunosorbent assay (ELISA). Statistics are as described in (B).

## SnCs have an altered response to SARS-CoV-2 spike protein

Viral entry through cell surface receptors and dampening of host antiviral gene expression are critical steps in successful infections and virus propagation (*18*). The spike 1 (S1) glycoprotein of SARS-CoV-2, antibodies against which are currently being tested in clinical trials (NCT04425629), mediates entry into host cells through binding to angiotensin-converting enzyme 2 (ACE-2), resulting in elevated nuclear factor κB (NF-κB) signaling and inflammatory cytokine production (*19*, *20*). Endothelial cells can be infected directly by SARS-CoV-2, leading to amplification of inflammation with subustantial changes in endothelial morphology and disruption of intercellular junctions (*21*).

**Fig. 2:**
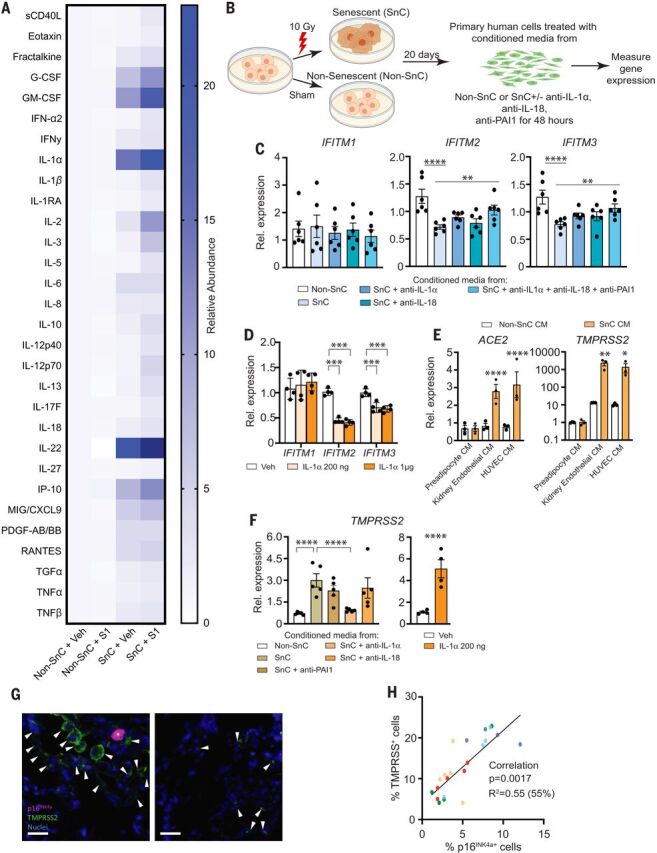
The SARS-CoV2 spike protein-1 (S1) exacerbates the secretory phenotype of senescent human endothelial cells, decreasing viral defenses and elevating viral entry/processing gene expression. (**A**) Primary human kidney endothelial cells (*n* = 9 biological replicates) were induced to undergo senescence with 10 Gy of ionizing radiation (SnC) or not (non-SnC) then treated with 500 ng recombinant S1 or PBS vehicle for 24 hours. Thirty SASP-related proteins were measured in the conditioned media (CM) by means of Luminex xMAP technology. Relative abundance induced by S1, normalized to vehicle treated non-SnCs (non-SnC + Veh), is illustrated in the heat map. A mixed effects model was used to test the effect of S1, senescence, and their interaction, taking into account duplicate measures within a subject for each protein as well as the composite score. Margin effects of SnCs in the treatment group also were tested under the mixed-effects model framework. Overall, the effect of S1 on SnCs was significantly more pronounced than on non-SnCs (composite score change *P* < 0.0089; mean values and *P* values for each cytokine are in table S2). (**B**) Schematic of experiments in (C), (E), and (F). Primary human cells were induced to undergo senescence with 10 Gy of ionizing radiation (SnC) or not (non-SnC). Twenty days later, CM was collected (*n* = 4 biological replicates) and used to treat non-SnCs (*n* = 4 biological replicates) either with or without neutralizing antibodies to IL-1α, IL-18, and PAI-1 (alone or in combination) for 48 hours, then RNA was isolated to measure expression of genes related to SARS-CoV-2 pathogenesis by means of quantitative PCR. Expression in cells treated with SnC CM was normalized to cells treated with non-SnC CM. Data are displayed as mean ± SEM, mixed-effects model. *P < 0.05 **P < 0.01, ***P < 0.001, ****P < 0.0001. (**C**) *IFITM* expression in human kidney endothelial cells treated with CM from SnC versus non-SnC human kidney endothelial cells. (**D**) *IFITM* expression in human kidney endothelial cells exposed to two concentrations of IL-1α (*n* = 4 biological replicates). Expression was normalized to vehicle-treated samples. (**E**) Gene expression in human lung epithelial cells treated with CM from SnC versus non-SnC preadipocytes, HUVECs, or kidney endothelial cells. (**F**) *TMPRSS2* expression in human kidney endothelial cells treated with CM from SnC versus non-SnC kidney endothelial cells with or without neutralizing antibodies or human kidney endothelial cells with recombinant IL-1α for 48 hours. (**G**) Human lung biopsies acquired for clinical indications of focal, noninfectious causes from elderly patients were stained for TMPRSS2, p16^INK4a^, and 4′,6-diamidino-2-phenylindole (DAPI) to detect nuclei (*n* = 5 subjects). Representative images are shown. Scale bar, 20 μm. (**H**) TMPRSS2^+^, p16^INK4a+^, and total nuclei were counted and expressed as a function of total nuclei in each field. TMPRSS2^+^ and p16^INK4a+^ cells/field were tightly linked (*P* < 0.0001; partial Pearson correlation). Each color series of dots indicates replicates from a single subject.

To address specifically how the SARS-CoV-2 PAMPs affect SnCs, senescent human kidney endothelial cells (fig. S4) were treated with pyrogen-free recombinant S1 protein. Exposing endothelial SnCs to S1 for 24 hours significantly increased secretion of the majority of endothelial SASP factors measured in the conditioned media (composite score *P* < 0.0089 comparing SnCs to non-SnCs) (Fig. 2A and table S2). Similar albeit less distinctive results were obtained by using kidney endothelial cells in which senescence was induced through replication rather than radiation (fig. S5 and table S3). In addition, treatment of human subcutaneous adipocyte progenitor SnCs with S1 increased expression of the key preadipocyte SASP factors, *IL1*α and *IL1*β, at the mRNA level (fig. S6). Consistent with the LPS data, these data suggest that S1 is a PAMP that can trigger a hyperinflammatory state in SnCs, possibly through stimulation of a Toll-like receptor (TLR) (*22*, *23*), with the inflammatory profile differing among types of SnCs.

Inflammatory SASP factors contribute to clearing pathogens. However, certain inflammatory/SASP factors released by senescent human lung cell types—including IL-1α, IL-1β, IL-6, MCP-1, TNFα, and MMP-1—are central to the pathological cytokine storm seen in some COVID-19 patients (*4*, *5*, *24*–*33*). Initially, to determine whether the SASP affects the response of human endothelial cells to pathogen exposure, nonsenescent primary kidney endothelial cells were exposed to conditioned media (CM) from SnCs or non-SnCs (Fig. 2B). The CM from SnC endothelial cells significantly reduced expression of the key viral defense genes *IFITM2* and *IFITM3* (Fig. 2C). IL-1α is a natural pyrogen as well as a master up-stream regulator of the senescence-associated IL-6/IL-8 cytokine network (*34*). It is increased in COVID-19 patients (*35*), increased in SnCs treated with S1 (fig. S6), and increased in LPS-treated mice (Fig. 1B and figs. S2 and S3). Directly treating nonsenescent primary human endothelial cells with IL-1α significantly reduced expression of *IFITM2* and *IFITM3* (Fig. 2D). Suppressing the SASP factors IL-18, PAI-1, and IL-1α by pretreating the CM from SnCs with neutralizing antibodies against these proteins partially restored *IFITM2* and *IFITM3* expression (Fig. 2C). These data support the conclusion that the SASP from preexisting SnCs could exacerbate SARS-CoV-2 infection of nonsenescent human endothelial cells.

Next, we examined the impact of the SASP on human lung epithelial cells, another target cell type in COVID-19. Treating nonsenescent primary human lung epithelial cells with CM from senescent human preadipocytes, kidney endothelial cells, or human umbilical vein endothelial cells (HUVECs) significantly increased expression of the SARS-CoV-2 viral entry genes *ACE2* and *TMPRSS2* (Fig. 2E). Similarly, treating nonsenescent human primary kidney endothelial cells with CM from SnCs induced expression of *TMPRSS2* (Fig. 2E). Adding neutralizing antibodies against IL-1α to the CM from SnC kidney endothelial cells reduced expression of *TMPRSS2*, whereas antibodies against IL-18 did not (Fig. 2F). Treating nonsenescent human primary endothelial cells directly with IL-1α increased *TMPRSS2* expression fivefold (Fig. 2F), and IL-1α treatment of nonsenescent human lung epithelial cells increased both *ACE2* and *TMPRSS2* expression twofold (fig. S7A). Treating nonsenescent human kidney endothelial cells with IL-1α also significantly increased expression of *IL6*, *IL8*, *IP10*, and *MCP1* (fig. S7B). In addition, although *ACE2* and *TMPRSS2* were not up-regulated in senescent human preadipocytes (fig. S7C) in which these genes are not normally expressed, *TMPRSS2* was up-regulated in senescent human endothelial cells (fig. S7D). Consistent with these in vitro results, in healthy human lung tissue resected from five elderly patients for clinical indications of focal, noninfectious causes, there were more TMPRSS2^+^ cells adjacent to p16^INK4a+^ cells as detected with immunofluorescence, with the abundance of p16^INK4a+^ cells correlating with TMPRSS2^+^ cell abundance (Fig. 2, G and H). Collectively, these data further support the conclusion that SnCs could promote SARS-CoV-2 pathogenesis by decreasing viral defenses and increasing expression of viral entry proteins in neighboring non-SnCs through amplified secretion of SASP factors.

## Old mice are hypersensitive to pathogen exposure, including β-coronavirus infection

**Fig. 3:**
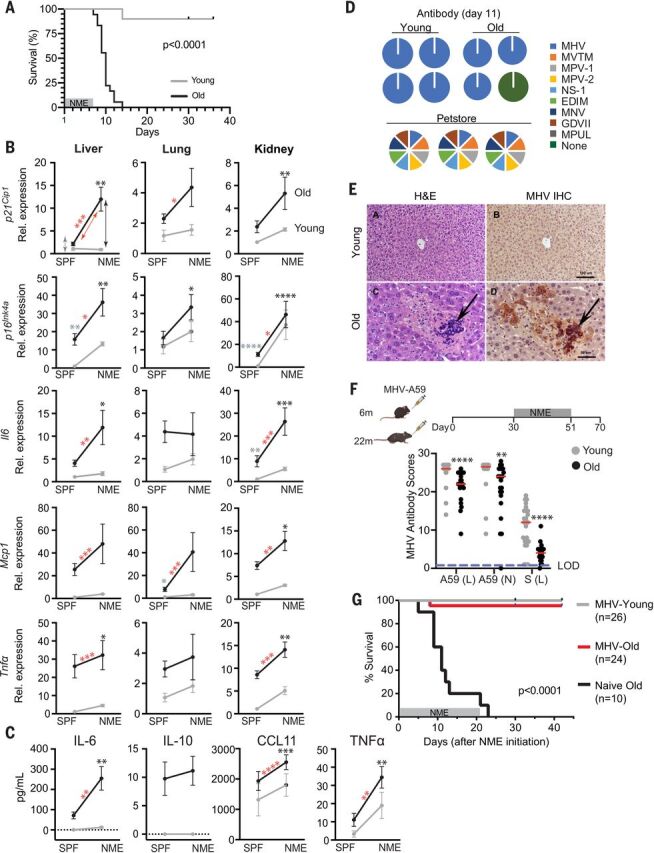
Old mice are vulnerable to a NME that includes acute mouse β-coronavirus infection. (**A**) Young (3-month-old) and old (20- to 24-month-old) WT mice were exposed to NME bedding produced from pet store mice for 7 days. Survival was monitored for 35 days after initiation of NME (*n* = 10 young; *n* = 18 old). Log-rank (Mantel Cox) test. (**B**) Gene expression in three tissues of SPF or NME (6- to 7-day exposure) young and old mice (*n* = 3 young SPF; *n* = 5 old SPF; *n* = 14 young NME; *n* = 13 old NME) measured with quantitative PCR. Expression was normalized to young SPF mice. Means ± SEM, two-way ANOVA and post hoc comparison Tukey’s honestly significant difference were used to compare the two animal cohorts within a treatment group. *P < 0.05, **P < 0.01, ***P < 0.001, ****P < 0.0001. Arrows and asterisks: gray, SPF old versus young; black, NME old versus young; red, old SPF versus old NME. (**C**) Serum cytokine levels in young and old mice (*n* = 3 young SPF; *n* = 5 old SPF; *n* = 19 young NME; *n* = 17 old NME) measured with ELISA at day 5 after NME. Statistics are as described in (B). (**D**) Serology to detect antibodies against microbes in NME bedding. (Right) The mouse pathogens commonly tested for by Charles River Laboratory to define SPF housing. The pie charts illustrate the exposures detected in individual young and old mice (*n* = 24 young; *n* = 21 to 23 old) day 11 after initiation of NME. Serology of pet store mice is illustrated below. (**E**) Representative images of hematoxylin and eosin (H&E) staining or MHV immunohistochemistry in liver sections from young and old mice exposed to NME. (**F**) (Top) Schematic to illustrate the experimental design. Young (6-month-old) or old (22-month-old) female mice were inoculated with a sublethal dose of MHV. Thirty days later, naïve and inoculated mice were exposed to NME bedding for 3 weeks. (Bottom) Serum antibodies against three different MHV antigens measured 21 days after MHV inoculation and reported as relative scores. The dotted line indicates the limit of detection (LOD). Means ± SEM, unpaired two-tailed Student’s *t* test. **P < 0.01, ****P < 0.0001. (**G**) Survival of MHV-inoculated and naïve mice measured for 42 days after initiation of NME. Log-rank (Mantel Cox) test.

To investigate the role of SnCs in driving adverse outcomes upon infection in vivo, we exploited an experimental paradigm developed to study the response of laboratory [specified-pathogen free (SPF)] mice to infection with common mouse microbes, creating what is termed a “normal microbial experience” (NME) (*36*–*38*). Experimental mice are exposed to pathogens through cohousing with pet-store mice or through exposure to their dirty bedding. NME exposure for many months rarely compromises the viability of young mice (89% survival across all experiments) (Fig. 3A) (*36*–*38*). By contrast, exposing old mice (20+ months of age) to the same NME rapidly caused nearly 100% lethality in <2 weeks and in both sexes (Fig. 3A and fig. S8A). In mice euthanized on day 6 or 7 after NME exposure, expression of senescence markers (*p21^Cip1^* and *p16^Ink4a^*) and SASP factors (*Il6*, *Mcp1*, and *Tnf*α) in liver, kidney, and to a lesser extent in lung were increased in old NME mice compared with old SPF, young SPF, or young NME mice (Fig. 3B). In addition, there was an increase in infiltration of CD45^+^ cells into the liver by day 6 or 7 after NME exposure in both young and aged mice (fig. S8B). The percent of infiltrating immune cells was significantly higher in aged mice than in young animals. These results are consistent with spread of senescence and inflammation after pathogen exposure. In addition, there was a significant increase in SASP-related inflammatory cytokines (IL-6, IL-10, EOTAXIN/CCL11, and TNFα) in the serum of old NME mice compared with young NME mice (Fig. 3C), which is consistent with preexisting SnCs creating an environment that contributes to hyperinflammation upon infection.

Several viruses were detected in saliva and fecal pellets from the NME mice a week after exposure to pet store mice, including the β-coronavirus mouse hepatitis virus (MHV), a virus in the same family as SARS-CoV-1 and -2 (table S4). However, by day 11, when the majority of old mice had succumbed to infection, NME mice were serologically positive for MHV but not the other pathogens carried by pet store mice (Fig. 3D). Histopathology indicated that old but not young mice had evidence of active MHV infection, manifested as multifocal necrotizing hepatitis and the presence of MHV-specific syncytial cells within areas of necrosis (Fig. 3E). In addition, MHV-induced syncytial cells were observed among epithelial cells in the small and large intestines of aged mice (fig. S8C). These findings are consistent with active infection in aged animals, in contrast to rapid clearance in the young animals.

To determine whether MHV infection contributes to NME-mediated mortality in old mice, young and old mice were directly infected with a sublethal dose of MHV (strain A59) before NME exposure (Fig. 3F). Old mice challenged with MHV generated a reduced antibody response compared with young mice (Fig. 3F). However, MHV immunization prevented death of the old mice after NME exposure, although the animals were infected with multiple other viruses (table S5), whereas naïve, old mice succumbed (Fig. 3G). This provides compelling evidence that the β-coronavirus MHV is the primary driver of mortality in old mice in the NME paradigm.

## Senolytics reduce senescence, inflammation, and mortality after pathogen exposure

To determine whether drugs that induce apoptosis specifically of SnCs, termed senolytics, reduce the mortality of old mice acutely infected with pathogens, we tested fisetin, a natural flavonoid found in many fruits and vegetables (*39*, *40*) that we established as senolytic (*14*, *41*). fisetin improves tissue homeostasis, reverses age-related tissue damage, and extends median life span of mice, even when administered late in life, with no observable adverse effects (*14*, *41*).

**Fig. 4:**
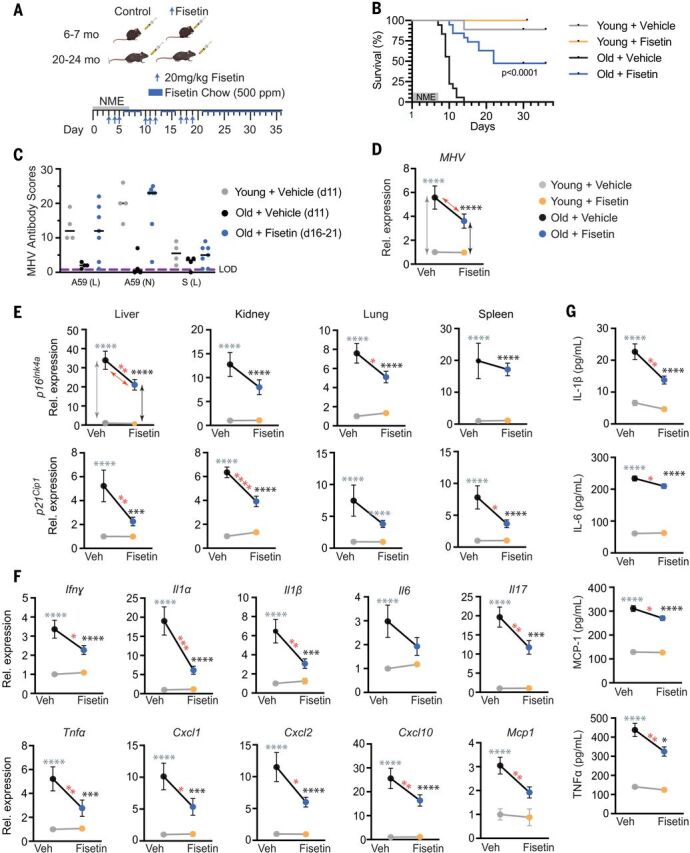
Treatment with the senolytic fisetin decreases mortality in NME-exposed old mice. (**A**) Schematic of the experiment. Young (6 to 7 months) and old (20 to 24 months) mice were exposed to NME bedding containing mouse β-coronavirus MHV for 7 days. Mice were treated with 20 mg/kg/day Fisetin or vehicle only by means of oral gavage daily for 3 consecutive days starting on day 3 after initiation of NME. The 3 days of treatment were repeated (3 days on, 4 days off) for 3 weeks. Animals were also fed standard chow with Fisetin added (500 ppm) ad libitum after initiation of treatment. (**B**) Survival was measured for 36 days after initiation of NME (*n* = 9 young + vehicle; *n* = 5 young + Fisetin; *n* = 18 old + vehicle; *n* = 19 old + Fisetin). Log-rank (Mantel Cox) test. *P* < 0.0001 for old mice ± Fisetin. (**C**) Relative MHV antibody score in young and old mice in (B) on the indicated day after initiation of NME. (**D** to **G**) Young (2-month-old) and old (20-month-old) mice were exposed to NME bedding ± treatment with Fisetin as described in (A). On days 8 to 9 after initiation of NME, animals were euthanized, and tissues collected for measuring gene expression (*n* = 10 young + vehicle; *n* = 8 to 10 young + Fisetin; *n* = 10 to 11 old + vehicle; *n* = 13 old + Fisetin). All expression data were normalized to young mice treated with vehicle. Data are displayed as means ± SEM, two-way ANOVA and post-hoc comparison Tukey’s honestly significant difference used to compare the two animal cohorts within a treatment group. Arrows and asterisks: gray, vehicle-treated old versus young; black, Fisetin-treated old versus young; red, old ± Fisetin. *P < 0.05, **P < 0.01, ***P < 0.001, ****P < 0.0001. (D) MHV mRNA was quantified by means of quantitative PCR in fecal pellets collected from individual animals in (C). (E) Quantification of *p16^Ink4a^* and *p21^Cip1^* mRNA in four tissues. (F) Quantification of SASP factor mRNA in liver. Data on other genes and tissues are available in fig. S9. (G) SASP protein levels in the liver measured with ELISA.

Old mice were exposed to NME for 1 week starting on day 0 and were then treated with 20 mg/kg fisetin by means of oral gavage on days 3 to 5, 10 to 12, and 17 to 19 after pathogen exposure (Fig. 4A), with no evidence of adverse effects. In between fisetin dosing, the mice were on a maintenance dose of fisetin [500 parts per million (ppm) Fisetin in chow ad libitum]. Consistent with our previous results (Fig. 3A), 100% of the old mice in the vehicle control groups died within 2 weeks (Fig. 4B, sexes combined, and fig. S9A, graphed by sex). However, 64% of the fisetin-treated male mice and 22% of the female mice survived long-term with a significant extension of overall life span for both sexes. Whether there is a true sex difference in the effect of fisetin on survival needs to be explored further because the ages of the old male and female mice were not identical.

On day 11 after NME, relative levels of antibodies against MHV were dramatically lower in the old than young mice (Fig. 4C), which is consistent with the premature death of the old mice. However, in old mice treated with fisetin, antibodies against MHV were increased to youthful levels by day 16. All mice exposed to NME were confirmed MHV-positive by means of reverse transcription polymerase chain reaction (RT-PCR) at 8 days after exposure (Fig. 4D). The old mice had significantly more viral mRNA than that of young mice (Fig. 4D), which is consistent with impaired immune responses in aged organisms (Fig. 3F) and impaired viral defenses because of SnCs (Fig. 2C). However, a short duration of fisetin treatment initiated 3 days after NME exposure tended to reduce the viral mRNA burden in old mice (*P* = 0.09) (Fig. 4D).

To evaluate how fisetin mediates its protective effects on NME-induced mortality in aged mice, we measured senescence and SASP markers before death. Cellular senescence markers (*p16^Ink4a^* or *p21^Cip1^*) were reduced in the liver, kidney, lung, and spleen of the old fisetin-treated NME mice compared with old mice receiving vehicle only (Fig. 4E). Furthermore, expression of multiple SASP inflammatory factors—including *Ifn*γ, *Il1*α, *Il1*β, *Il6*, *Il17*, *Tnf*α, *Cxcl1*, *Cxcl2*, *Cxcl10*, *Mcp1*, *Mip1*, *Pai1*, *Pai2*, *Il2*, and *Il7*—was reduced to varying extents in the same tissues (Fig. 4F and fig. S9B). Similarly, the levels of circulating IL-1β, IL-6, MCP-1, and TNFα were reduced after fisetin treatment (Fig. 4G). Thus, although the old mice were MHV-infected, fisetin reduced senescence, the SASP, and inflammation after infection and prolonged survival, enabling an improved antibody response to the virus.

## Senolysis contributes to improved outcomes in old mice exposed to pathogens

**Fig. 5:**
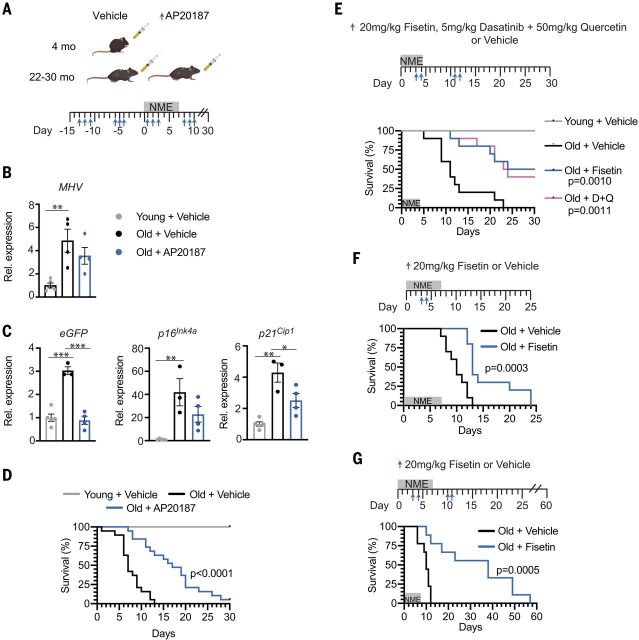
Pharmacologic and genetic ablation of SnCs reduces mortality in old mice exposed to NME. (**A**) Schematic diagram of the experimental design for (B) to (D). Young (4-month-old) and old (22- to 30-month-old) male and female *INK-ATTAC* mice were treated with vehicle or AP20187 (*n* = 10 young; *n* = 19 old + vehicle; *n* = 19 old + AP20187) to dimerize FKBP-caspase-8 fusion protein expressed in *p16^Ink4a+^* cells to kill SnC selectively. AP20187 (10 mg/kg) or vehicle was administered intraperitoneally daily for 3 days starting 2 weeks before initiating NME and ending 1 week after (3 days on and 4 days). NME was started on day 0 and lasted 1 week. Mice housed in SPF conditions were used as controls. Tissues were collected 7 days after initiation of NME in another cohort of male animals for molecular analysis (*n* = 5 young ; *n* = 3 or 4 old + vehicle; *n* = 4 old + AP20187). (**B**) Quantification of MHV mRNA in fecal pellets isolated from individual mice. Means ± SEM, one-way ANOVA with Tukey’s test. **P < 0.01. (**C**) Quantification of *eGFP* (a reporter of *p16^Ink4a^* expression in the *INK-ATTAC* construct), *p16^Ink4a^*, and *p21^Cip1^* mRNA in the kidney of mice in (B). All expression data were normalized to young mice treated with vehicle. Means ± SEM, one-way ANOVA. *P < 0.05, **P < 0.01, ***P < 0.001. (**D**) Survival of male and female mice measured for 30 days after initiation of NME. Log-rank (Mantel Cox) test. (**E**) Young (2-month-old, *n* = 5) and old (22-month-old, *n* = 10/group) female mice were exposed to NME bedding for 4 days. Beginning on day 3, mice were treated with 20 mg/kg Fisetin or 5 mg/kg Dasatinib plus 50 mg/kg Quercetin at days 3, 4, 11, and 12 by means of oral gavage, or with vehicle only. Survival was measured for 30 days after initiation of NME. Log-rank (Mantel Cox) test. (**F**) Survival curves for 20-month-old WT female mice (*n* = 10/treatment group) treated with 20 mg/kg Fisetin or vehicle by oral gavage on days 3 and 4 after initiation of NME exposure. Log-rank (Mantel Cox) test. (**G**) Survival of 22-month-old WT female mice (*n* = 9/treatment group) treated with 20 mg/kg Fisetin or vehicle only by oral gavage at days 3, 4, 10, and 11 after NME exposure monitored out to 60 days after exposure. Log-rank (Mantel Cox) test.

To determine whether the mechanism of action of fisetin in suppressing adverse outcomes upon viral infection includes senolysis, two approaches were taken. First, *INK-ATTAC* mice were studied under NME conditions to enable genetic ablation of *p16^Ink4a^*-expressing SnCs (*42*). *INK-ATTAC* mice express a caspase 8-FKBP fusion protein, *ATTAC* (*43*), from the *p16^Ink4a^* promoter. Old *INK-ATTAC* mice (>24 months) were treated with AP20187 to drive dimerization of FKBP, activation of caspase-8, and apoptosis of *p16^Ink4a^*-expressing cells (3 days per week × 2 weeks), before exposure to NME and then weekly after NME (Fig. 5A). Both control and AP20187-treated mice were positive for MHV RNA at day 8 after NME exposure (Fig. 5B). AP20187 treatment reduced the expression of the SnC markers *p16^Ink4a^* and *p21^Cip-1^* and of enhanced green fluorescent protein (*eGFP*), which is also driven by the *p16^Ink4a^* promoter in *INK-ATTAC* mice after NME exposure (Fig. 5C), as well as certain inflammatory/SASP genes in kidney, liver, brain, pancreas, and/or colon (fig. S10). AP20187 treatment significantly delayed NME-induced mortality in both male and female aged mice (Fig. 5D and fig. S10A), providing evidence that senolysis improves outcomes in aged organisms acutely exposed to pathogens. The level of MHV RNA also trended down after AP20187 treatment (Fig. 5B), which is consistent with the results with fisetin treatment.

Second, we tested a different well-established senolytic cocktail, Dasatinib plus Quercetin (D+Q) (*12*, *13*), and directly compared it with fisetin in the same survival experiment. D+Q or fisetin was administered to aged female mice at days 3 or 4 then 11 or 12, respectively, after initiation of NME (Fig. 5E). As expected, whereas 100% of the old, vehicle-treated mice succumbed to infection, ~50% of the old mice treated with D+Q or fisetin survived (Fig. 5E). The similarity in survival curves between the two treatment groups is notable. This combination of genetic and pharmacologic studies provides strong support for the conclusion that clearing SnCs in old organisms contributes to improved outcomes upon acute exposure to viral pathogens.

Last, to determine whether pretreating old mice with fisetin after infection could prevent adverse outcomes, old WT mice were treated with a single round of high-dose fisetin (20 mg/kg/day for 2 consecutive days beginning 3 days before NME exposure), followed by low-dose fisetin after infection (fig. S11A). This suppressed mortality in both male and female mice by 40% (fig. S11, A and B). Additionally, antibodies against MHV were detected in fisetin-treated mice on days 16 and 21 (fig. S11C), a time by which all vehicle-treated old mice had died (fig. S11A). To evaluate whether a shorter regimen of senolytic therapy could improve outcomes in old NME mice, animals were given after NME exposure two doses of fisetin once (days 3 and 4) (Fig. 5F) or twice (days 3 and 4 then days 10 and 11) (Fig. 5G). These short-course treatments, in the absence of continuous exposure to fisetin through chow, were sufficient to delay mortality significantly (Fig. 5, F and G). Because Fisetin has an elimination half-life of less than 5 hours (*44*), these data are consistent with a “hit-and-run” mechanism, in which fisetin is acting as a senolytic, reducing overall SnC burden, rather than being required to be present constantly to engage with a molecular target to confer benefit. The data also reveal that fisetin can be administered in a pulsatile fashion before or after viral infection to reduce mortality of old organisms.

## Discussion

**Fig. 6:**
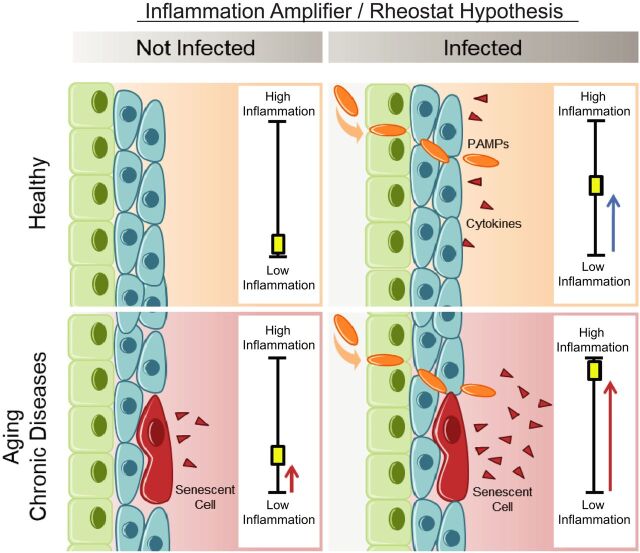
SASP Amplifier/Rheostat hypothesis. Schematic of the hypothesis generated from these data and tested herein. SnC amplified the response to PAMPs in vitro and in vivo, resulting in increased production of pro-inflammatory cytokines and chemokines. This could exacerbate acute systemic inflammatory responses and cytokine release by innate immune cells and amplify the spread of senescence. This model could explain the increased risk of cytokine storm during COVID-19 or other infections and adverse outcomes observed in the elderly or those with chronic conditions associated with an increased burden of SnC (obesity, diabetes, chronic lung or kidney disease, or cardiovascular disease).

Our study demonstrates that SnCs are primed to respond to PAMPs by expressing and secreting even higher levels of inflammatory SASP factors than that in healthy cells. These PAMPs include the SARS-CoV-2 S1, which exacerbate the SASP of human SnCs and, in turn, reduce innate viral defenses and increase expression of SARS-CoV-2 viral entry proteins in nonsenescent human lung cells and tissue. On the basis of these observations, we formulated the “Amplifier/Rheostat” hypothesis, in which PAMPs, such as SARS-CoV-2 S1 viral antigen, cause a shift in the SASP of preexisting SnCs into a more highly inflammatory, profibrotic SASP (Fig. 6). The amplified SASP factors include cytokines and chemokines, such as IL-1α, that exacerbate systemic inflammation and drive secondary senescence. These secondary SnCs can then (i) further exacerbate and prolong inflammation, (ii) reduce viral defenses in non-SnCs, (iii) facilitate viral entry in non-SnCs, (iv) attenuate or delay recovery, (v) contribute to persistent frailty, (vi) cause tissue fibrosis, and (vii) contribute to hyper-inflammation and multi-organ failure.

Our Amplifier/Rheostat hypothesis is supported by in vivo results, first by using acute LPS treatment and subsequently by exposing old mice to a NME, which included a mouse β-coronavirus related to SARS-CoV-2. We demonstrate that the SnC burden in old mice confers, at least in part, the reduced resilience, increased inflammation, impaired immune response, and mortality observed in old male and female mice exposed to new viral pathogens. Both the pharmacological (such as senolytics fisetin or D+Q) and genetic (*INK-ATTAC*) clearance of SnCs yielded significant delay or, in the case of the former, reduction in mortality in both old male and female mice. Adverse outcomes were attenuated when the senolytic fisetin was administered either before (a preventative measure) or after (a therapeutic intervention) NME exposure. The senolytics fisetin and D+Q were more effective at delaying mortality than were genetic ablation of SnCs in the *INK-ATTAC* mice (Fig. 5, F versus D), which is consistent with the latter only removing SnCs that express high levels of *p16^Ink4a^* and not p16-low or -negative SnCs. However, subtle differences in fomite bedding make it difficult to compare life span data between experiments.

Although the NME paradigm does not directly model SARS-CoV-2 infection, NME exposure involves transmission of multiple common community-acquired mouse infectious agents. Among these is the β-coronavirus MHV, which is an enteric virus transmitted by oral or fecal spread rather than respiratory droplets. Even though MHV infects hepatocytes to a greater extent than pulmonary tissue, we did find evidence of inflammation in the lung, spleen, liver, gastrointestinal tract, and kidney, similar to that in COVID-19 patients. MHV was a primary viral pathogen transferred by the NME as evidenced with serology, quantitative RT-PCR, and liver histopathology and caused severe disease in aged but not young, mice. Furthermore, MHV immunization conferred protection from NME-induced mortality, indicating an essential role for the β-coronavirus in the mortality of old mice. The NME model does accurately reflect the dramatic response of naïve organisms to a novel β-coronavirus, the age disparity in outcomes observed in COVID-19 patients, the hyperinflammation elicited in some hosts, and the common experience of opportunistic infections contributing to disease severity and mortality.

SnC burden is increased in old and young mice exposed to NME (Fig. 3B) and, if it persists, could lead to additional comorbidities. However, the magnitude of senescence in young animals appears not to reach a threshold that compromises survival. Thus, it is possible that senolytic treatment could be beneficial to COVID-19 survivors for improving long-term outcomes and suggests that monitoring expression of senescence markers in this patient population would be advantageous. Moreover, it was not necessary to reduce senescence markers to the level of young individuals to dramatically improve survival. This supports the possibility that there is a threshold beyond which senescent cell burden is deleterious (*13*, *45*) and illustrates that unlike for cancer cells, not every SnC needs to be eliminated to have a beneficial effect.

A high SnC burden in the elderly or those with chronic diseases such as diabetes, obesity, hypertension, or chronic lung disease likely can interfere with the ability of the immune system to induce a strong B and T cell response to new antigens. We found that intermittent senolytic treatment improved the development of an antibody-against-MHV response. This could be because the old mice survive long enough to mount a healthy response analogous to younger mice, or because dampening the SASP and inflammation improved immune cell function, or both. However, our preclinical data suggest that senolytics could improve the response of the elderly to vaccines for SARS-CoV-2 andother viral pathogens.

The immediate implication of these studies is that senolytics could have clinical application for attenuating mortality and other adverse outcomes in the elderly and those with comorbidities who become infected with SARS-CoV-2. Furthermore, on the basis of our findings in LPS-treated SnCs and aged mice, senolytics may be of potential therapeutic use for elderly persons stricken by bacterial infections. In addition, our data support the view that targeting pillars of aging and, in particular, cellular senescence can improve resilience of the elderly in the face of viral pathogens. This strongly supports the Geroscience hypothesis that targeting fundamental aging mechanisms can improve health span in the elderly and implies that targeting other pillars of aging might also alleviate morbidity from viral infection. Thus, for the COVID-19 pandemic as well as future pandemics, rapalogs, glucocorticoids, and metformin, all of which inhibit the SASP, might lessen SARS-CoV-2 cytokine storm and improve outcomes (*46*–*48*). However, unlike senolytics, some of these drugs may need to be administered continuously or at least more frequently, adding to off-target and side effects, especially in elderly patients with comorbidities and polypharmacy. The SASP Amplifier hypothesis, supported by data presented here, led to the initiation of a clinical trial (NCT04476953) to test whether fisetin prevents disease progression in hospitalized older COVID-19 patients. A similar but larger multisite trial to test fisetin in elderly COVID-19 patients in nursing homes (NCT04537299) also has been initiated. Last, although there are now vaccines for SARS-CoV-2 being distributed, it will take a long time for a significant percentage of the world’s population to be vaccinated. Even if the 95% effectiveness rate of the vaccines in healthy populations is borne out in elderly nursing home residents, still at least 1 out of 20 vaccinated elderly residents is anticipated to become infected by COVID-19 and will need treatment, potentially with senolytics and antivirals.

## Material and methods

### Animals

Wild-type C57BL/6 (young = 2 to 7 months of age; old = 20 months of age or older) mice were bred at the University of Minnesota or Mayo Clinic, purchased from Charles River (Wilmington, MA), Jackson Laboratory (Bar Harbor, ME), or received from the Aging Rodent Colony at the National Institute of Aging (Baltimore, MD). C57BL/6:FVB mice and *Ercc1*^−/∆^ mice were bred in the Niedernhofer laboratory at the University of Minnesota as previously described (*49*). The generation and characterization of the *INK-ATTAC* transgenic mouse line has been described (*42*). J.L.K., T.T., J. M. van Deursen, and D. J. Baker (all Mayo Clinic) designed the *INK-ATTAC* strategy. Pet store mice were purchased from local pet stores in the Minneapolis-St. Paul, MN metropolitan area. All mice were housed in AALAC-approved animal facilities at the University of Minnesota (BSL-1/-2 for SPF mice and BSL-3 for exposure to a natural microbial experience) or Mayo Clinic. Mice were randomly assigned to control or experimental groups based on weight and appearance. Experimental procedures were approved by the University of Minnesota and Mayo Clinic Institutional Animal Care and Use Committees and performed following the Office of Laboratory Animal Welfare guidelines and PHS Policy on Use of Laboratory Animals.

### Mouse experiments

LPS challenge: WT mice were injected intraperitoneally with either LPS (500 ng/kg) or vehicle (PBS). Animals were euthanized 24 hours post-injection and tissues collected. Total RNA was isolated from kidney and liver for the analysis of senescence and SASP marker expression by quantitative PCR (qPCR) using the ∆∆Ct method, with *Gapdh* serving as a housekeeping control. Serum levels of IL-6, MCP-1, TNF⍺ were analyzed by ELISA.

### Normal microbial experience (NME)

Immune-experienced mice were obtained from different vendors around Minneapolis, MN and were used as carriers of transmissible pathogens (hereafter called pet mice). Laboratory strains of mice were either directly cohoused with pet mice (*37*) or were housed on soiled bedding (totaling 150 to 300 cm^3^/cage) that were collected from cages of pet store mice after 1 week of housing (fomites). Mice were housed in AALAC-approved ABSL3 animal facilities at the University of Minnesota and were monitored daily.

### Senolytic preparation and administration

Fisetin (Indofine Chemical) or Dasatinib (LC laboratories. Cat# D-337, Woburn, MA) and Quercetin (Sigma. cat#Q4951-10G, St. Louis, MO) were dissolved in vehicle (10% ethanol, 30% polyethylene glycol 60% phosal 50 pg). Mice were weighed and given Fisetin (20 mg/kg), D+Q (5mg/kg+50mg/kg respectively), or vehicle control alone by oral gavage as indicated. Fisetin (500 ppm) was compounded into mouse chow (standard mouse diet, Lab Diet 5053). AP20187 was purchased from Clontech (Mountain View, CA). Vehicle (10% ethanol, 30% polyethylene glycol 60% phosal 50 pg) or AP20187 dissolved in vehicle was injected IP (10 mg/kg).

### Tissue harvest

For RNA extraction, tissues were snap-frozen in liquid nitrogen and kept frozen until nucleic acid isolation. For histopathology, tissues were fixed in formalin and paraffin embedded.

### Serology and measurement of viral RNA

Serum was collected at the indicated times for antibody screening using EZ-spot followed by a multiplexed fluorometric immunoassay (Charles River). The screening panel includes: mouse hepatitis virus (MHV), Sendai virus, pneumonia virus of mice, minute virus of mice (MVM), mouse parvovirus type 1(MPV), mouse parvovirus type 2, mouse parvovirus-NS1, murine norovirus (MNV), Theiler’s murine encephalomyelitis virus (TMEV), reovirus, rotavirus EDIM, lymphocytic choriomeningitis virus, ectromelia virus, mouse adenovirus 1 and 2, mouse cytomegalovirus, polyoma virus, *Mycoplasma pulmonis*, *Enchephalitozoon cuniculi*, cilia-associated respiratory bacillus, and *Clostridium piliforme*. Relative serology scores for MHV antigens (recombinant A59-strain nucleocapsid protein, purified A-59 viral lysate, and purified S-strain viral lysate) were calculated by Charles River using median fluorescence index. Active pathogen infection was measured by PCR Rodent Infectious Agent (PRIA) array methods (Charles River) in samples collected from oral swabs or fecal material. This panel screened for MHV, MNV, MPV, MVM, *Rodentibacter heylii*, and *Helicobacter* species.

### Infection with MHV-A59

MHV-A59 virus was a kind gift of Dr. Stan Perlman (U of Iowa). Virus was propagated and tittered onto 17cl-1 cells. Doses of 6x10^5^-1x10^6^ PFU were delivered intranasally after briefly anesthetizing mice with Isofluorane.

### Histology

Formalin-fixed samples were processed and embedded in paraffin before being sectioned (4 μm) and stained with hematoxylin and eosin. MHV immunohistochemistry was performed using anti-MHV-JHM ascitic fluid (*50*) (gift from Dr. S. Compton, Yale University) and bound antibody was detected using the Dako ARK Peroxidase kit (Animal Research Kit, Code K3954) for detecting mouse primary antibodies (Agilent Dako, Carpinteria, CA). All histologic sections were analyzed by two board-certified veterinary pathologists (TWC, MGO’S).

### Serum cytokines and chemokines

Serum samples were analyzed by the Cytokine Reference Laboratory (CRL, University of Minnesota). Samples were analyzed for mouse specific IP10, IL-6, IL-1β, KC, IL-2, IFNγ, TNFα, LIX, MCP-1 MIP2, MIP1α, GMCSF, IL-10, and eotaxin using the multi-plex Luminex platform. Magnetic bead sets (cat. # MPTMAG-70K-14) were purchased from EMD Millipore (Burlington, MA). Proteins were measured according to the manufacturer’s instructions. The beads were read on a Luminex instrument (Bioplex 200). Samples were run in duplicate and values were interpolated from 5-parameter-fitted standard curves. Serum concentrations of IL-6 (Abcam cat.# ab222503) and MCP-1 (Raybiotech cat.# ELM-MCP1-CL1, Peachtree Corners, GA) in LPS- and vehicle-treated mice (Fig. 1 and figs. S2 and S3) were measured by single-analyte ELISAs with a Varioskan plate reader. Samples were run in duplicate.

### Measurement of cytokines and chemokines in liver

100 mg of tissue was homogenized in RIPA buffer and Complete Mini EDTA-free Protease Inhibitor and adjusted to 1 mg/mL. Samples were analyzed for mouse-specific IL-1β (Abcam cat.# ab197742, Cambridge, MA), IL-6 (Abcam cat.# ab222503), MCP-1 (Raybiotech cat.# ELM-MCP1-CL1), and TNF⍺ (Abcam cat.# ab208348) by ELISA.

### Cell culture

The kidney endothelial cells were from a female (21-week old) donor. Preadipocytes were isolated from abdominal subcutaneous fat biopsies obtained from 10 subjects (3 male; 7 female; median age 44.3 ± 9.2 years; BMI 44.6 ± 9.2) who underwent gastric bypass surgery. All subjects gave informed consent. The protocol was approved by the Mayo Clinic Institutional Review Board for Human Research. Cells were isolated, cultured, and made senescent as previously described (*12*). Human primary renal glomerular endothelial cells, ScienCell (Cat #4000, Carlsbad, CA), Human Small Airway Epithelial Cells (Cat# CC-2547, Lonza), and HUVECs (Lonza, Cat #CC-2519, Basel, Switzerland) were purchased and cultured following manufacturer’s instructions. Cells were treated with S1 antigen (RayBiotech, Cat #230-30162-100, Peachtree Corners, GA), LPS from E.coli O111:B4 purified by ion-exchange chromatography (Millipore Sigma, Cat#L3024), or antibodies for INF-α, for different durations as described in the manuscript. Briefly, senescent and non-senescent cells were treated with LPS for 3 hours. Cells were washed, and RNA was collected. Endothelial cells were treated with viral antigen for 24 hours, cells were washed and medium was replaced with fresh MEM containing 2% FBS for collecting conditioned medium (CM) after 24 hours. CM was filtered and cytokine and chemokine protein levels in CM were measured using Luminex xMAP technology. The multiplexing analysis was performed using the Luminex 100 system (Luminex, Austin, TX) by Eve Technologies Corp. (Calgary, Alberta, Canada). Human multiplex kits were from Millipore (Billerica, MA).

### Cell culture with conditioned media (CM) and recombinant IL-1α

Non-senescent human primary renal glomerular endothelial cells were co-cultured with CM from senescent or non-senescent human primary renal glomerular endothelial cells with or without neutralizing antibodies for IL-1α (Catalogue #7D4 Anti-hIL-1α-IgG, InvivoGen, San Diego, CA), IL-18 (Catalogue #PA5-47803, Thermo-Fisher, Waltham, MA), and PAI1 (Catalogue #MAB1786, R&D system, Minneapolis, MN) for 48 hours, and cells were collected for qPCR. Non-senescent human primary renal glomerular endothelial cells were co-cultured with recombinant human IL-1α protein (Catalogue #200-LA-010, R&D Systems, Minneapolis, MN) for 48 hours and cells were collected for qPCR.

### Lung biopsies

Methods for acquisition of human lung samples have been described previously (*51*, *52*). Following pre-surgical patient consent, lung specimens were obtained from resections incidental to thoracic surgery at Mayo Clinic Rochester for clinical indications of focal, non-infectious causes (typically lobectomies, rarely pneumonectomies, for focal cancers). Normal lung areas were identified with a pathologist (protocol approved by the Mayo Clinic Institutional Review Board). Samples were formalin-fixed and paraffin-embedded for immunostaining and histology. Subjects used in this study were one female, four males, age 65.4 ± 10 years old (mean ± SD). The remaining clinical information was de-identified prior to immunostaining.

### Immunostaining

Slides were rehydrated with xylene and decreasing concentrations of ethanol in water, blocked with endogenous peroxidase with 3% H_2_O_2_, and boiled for antigen retrieval in citrate buffer (pH 6.0). Sections were blocked with BSA 5% normal goat serum for 1 hour followed by overnight incubation with p16^INK4a^ mouse anti-human antibody (Roche Diagnostic, Clone E6H4, #705-4793, ‎Rotkreuz, Switzerland). After washing in TBST buffer, sections were incubated in goat anti-mouse HRP antibody (Invitrogen, Cat #31430, Carlsbad, CA) for 30 min in blocking buffer and stained with TSA Cy5 (Akoya Biosciences, Cat #NEL745001KT, Menlo Park, CA) for 10 min. Antibodies were stripped with a second round of antigen retrieval in citrate buffer (pH 6.0) following the TSA manufacturer’s protocol. After blocking steps, slides were incubated with rabbit anti-human TMPRSS2 antibody (#ab92323, Abcam) for 12 hours, washed, and incubated with secondary goat anti-rabbit HRP antibody (#31460, Invitrogen) for 30 min followed by 10 min of TSA FITC (Akoya Biosciences Cat #NEL741001KT). Slides were mounted in Prolong Gold anti-fade with DAPI (Thermo-Fisher Cat #P36935).

### Imaging

Imaging was performed using a Nikon T1 microscope (Nikon, Japan). A total of 10 images of the alveolar region of lungs were captured/slide. Background correction and intensity thresholding were defined using controls and applied to all samples using Advanced NIS Elements software (Nikon, Tokyo, Japan), with fine-adjustments for each subject’s background intensity. A total of 4 to 5 sections/slide with the best tissue integrity were selected for counting, and merged images were exported to ImageJ FIJI (*9*). We applied a centralized grid of 125 by 125 μm, generating 15 fields/section. TMPRSS2^+^ and p16^INK4a+^ cell counting markers were used to retrieve cell numbers in each square. The single-channel for DAPI was exported to ImageJ and the same 125 by 125 μm grid was applied, so nuclei could be counted in each square slice.

### RNA extraction

Tissues were snap-frozen after harvest. RNA was extracted using Trizol after homogenization in a bead beater. After homogenization, chloroform was added to each sample. Samples were centrifuged to separate the aqueous layer. RNA was purified using columns (PureLink RNA Mini Kit Cat#12183018A) according to the manufacturer’s instructions. Concentration and purity of samples were assayed using a Nanodrop spectrophotometer.

### RT-PCR and qPCR

Each cDNA sample was generated by reverse transcription using 1 to 2000 ng RNA and by following the recommended protocol from the manufacturer (High-capacity cDNA Reverse Transcription Kit; Thermo-Fisher Cat #4368813). A standard reverse transcription program was used (10 min at 25°C, 120 min at 37°C, 5 min at 85°C, held at 4°C). qPCR was performed using Taqman Fast Advanced Master Mix (Thermo-Fisher, Cat# numbers listed in supplemental table S3) and probes or PowerUp SYBR Green Master Mix and primer pairs. *Gapdh* was used as a control for gene expression analysis. Data were analyzed using the ∆∆Ct method.

### Statistical analysis

All data analyses were conducted in STATA 16.0 (College Station, TX: Stata Corp). All figures were plotted using Prism 9.0 (GraphPad) or R 3.6.2. *P* value ≤ 0.05 was considered statistically significant.

To test the normality of the distribution of original variables (for analysis of variance [ANOVA] and Student’s *t* test) or residuals (for linear mixed model), skewness and kurtosis tests were performed accordingly (*53*). If the normality assumption was rejected (i.e., *P* < 0.05), we used zero-skewness log transformation (*54*). Then we performed the normality test again. If it was still rejected, we used a Box-Cox power transformation. If neither of these worked, we used rank transformation (i.e., using the rank of the original variable) instead (*55*).

Student’s *t* test was used to compare the equality of means from two independent samples, while one-way ANOVA was used to compare means from multiple samples. Two-way ANOVA was used when there were two predictors and above. A linear mixed model was used if there was non-independence within individuals or experiments. Tukey HSD test was used for post-hoc multiple-comparison after one- or two-way ANOVA (*56*, *57*). In the case of mixed-effect models, “margins” command was used to calculate statistics from predictions of the fitted model at fixed values of some predictors (e.g., treatment and type of cells). Partial Pearson correlation and linear regression, both with adjustment for strain ID, were performed to examine the association between TMPRSS^2+^ and p16^INK4a+^. To assess whether the SASP factors changed as a group, we created a composite score for each individual, which is the average *z*-score of the involved factors and performed the mixed effect model using the composite score as the outcome to assess whether the SASP factors changes as a group varied across covariates (*58*)

$${\mathrm{Composite Score}}_{i}=\sum_{j=1}^{m_{i}}\frac{z_{ij}}{m_{i}}$$

where *z_ij_* is the *z*-score of transformed values (by either log-transformed, Box-Cox transformed, or rank transformed) of SASP factor *j* for individual *i*, respectively. *m_i_* is the number of observed factors for individual *i*. 

For survival data, Kaplan-Meier survival curves were used to describe the survival process, which was followed by a log-rank test for assessing the equality of survivor functions between groups if there was only one predictor, or a Cox proportional hazards model if there were two predictors. Interaction between two predictors (e.g., treatment and type of cells) was considered in the above analyses if the original design was a factorial one.

## Supplementary Materials

science.sciencemag.org/content/373/6552/eabe4832/suppl/DC1
Figs. S1 to S11 Tables S1 to S6 MDAR Reproducibility Checklist Data S1

## Appendix

View/request a protocol for this paper from *Bio-protocol*.
