# Supplementary material for: Senolytics reduce coronavirus-related mortality in old mice
Source: Science. Author manuscript; Available in PMC 2021 Nov 22. (PMC8607935; doi:10.1126/science.abe4832)
Supplement: Supplementary file 1 [file NIHMS1727091-supplement-Supplment.pdf]

science.sciencemag.org/cgi/content/full/science.abe4832/DC1

Supplementary Materials for

Senolytics reduce coronavirus-related mortality in old mice

Christina D. Camell†, Matthew J. Yousefzadeh†, Yi Zhu†, Larissa G. P. Langhi Prata†, Matthew A. Huggins, Mark Pierson, Lei Zhang, Ryan D. O'Kelly, Tamar Pirtskhalava, Pengcheng Xun, Keisuke Ejima, Ailing Xue, Utkarsh Tripathi, Jair Machado Espindola-Netto, Nino Giorgadze, Elizabeth J. Atkinson, Christina L. Inman, Kurt O. Johnson, Stephanie H. Cholensky, Timothy W. Carlson, Nathan K. LeBrasseur, Sundeep Khosla, M. Gerard O'Sullivan, David B. Allison, Stephen C. Jameson, Alexander Meves, Ming Li, Y. S. Prakash, Sergio E. Chiarella, Sara E. Hamilton*, Tamara Tchkonia*, Laura J. Niedernhofer*, James L. Kirkland*, Paul D. Robbins*

†These authors contributed equally to this work.
*Corresponding author. Email: probbins@umn.edu (P.D.R.); kirkland.james@mayo.edu (J.L.K.); lniedern@umn.edu (L.J.N.); tchkonia.tamar@mayo.edu (T.T.); hamil062@umn.edu (S.E.H.)

Published 8 June 2021 on *Science* First Release
DOI: 10.1126/science.abe4832

This PDF file includes:

Figs. S1 to S11
Tables S1 to S6

Other Supplementary Materials for this manuscript includes the following:
(available at science.sciencemag.org/cgi/content/full/science.abe4832/DC1)

MDAR Reproducibility Checklist (.pdf)

Supplemental Figures

Figure S1: Senescence-Associated Secretory Phenotype (SASP) factor expression is amplified by Pathogen-Activated Molecular Pattern (PAMP). As in Fig. 1A, human adipocyte progenitors isolated from subcutaneous fat biopsies (n=5 subjects) were induced to undergo senescence with 10 Gy of ionizing radiation (SnC) or not (non-SnC). Then cells were treated with different concentrations of LPS for 3 hr. Gene expression was measured by qPCR. mRNA expression was normalized to vehicle-treated non-SnC. Means ± SEM, mixed effects model. *p<0.05, **p<0.01, ***p<0.001, ****p<0.0001 for comparison SnC *vs.* non-SnC at the same concentration of LPS.

Figure S2: Senescence-Associated Secretory Phenotype (SASP) factors are amplified in kidneys of mice after Pathogen-Activated Molecular Pattern (PAMP) exposure. Kidney data from the same experiment as Figure 1B. Young (2-month-old) and old (26-month) mice were treated with PBS (n=5y and 5o) or LPS (n=4y and 3o) and tissues collected 24 hr later. RNA was isolated from kidney and gene expression measured by qPCR. Expression was normalized to vehicle-treated young mice. Means ± SEM, two-way ANOVA and *post-hoc* comparison Tukey's Honestly Significant Difference used to compare the two animal cohorts within a treatment group. Arrows and asterisks: grey=vehicle-treated old *vs.* young; black=LPS-treated old *vs.* young; red=old ± LPS. **p<0.01, ***p<0.001, ****p<0.0001.

Figure S3: Senescence-Associated Secretory Phenotype (SASP) factors are amplified in tissues of *Ercc1*$^{-/\Delta}$ progeroid mice after Pathogen-Activated Molecular Pattern (PAMP) exposure. Three-month-old progeroid *Ercc1*$^{-/\Delta}$ mice and WT littermates were treated with PBS or

LPS (n=4/group), then euthanized and tissues collected 24 hours later. **A.** RNA was isolated from kidney and liver and the following measured by qPCR: *p16^Ink4a^* and *p21^Cip1^*, *Il1a*, *Il1β, Il6*, *Mcp1*, and *Tnfα*. **B.** IL-6 and MCP-1 protein levels in serum from the same mice, measured by ELISA 24 hr later. Expression was normalized to vehicle-treated age-matched WT mice. Means ± SEM, two-way ANOVA and *post-hoc* comparison Tukey's Honestly Significant Difference used to compare the two animal cohorts within a treatment group. Arrows and asterisks: grey=vehicle-treated *Ercc1*$^{-/\Delta}$ *vs.* age-matched WT; black=LPS-treated *Ercc1*$^{-/\Delta}$ *vs.* age-matched WT; red=*Ercc1*$^{-/\Delta}$ ± LPS. **p<0.01, ***p<0.001, ****p<0.0001.

Figure S4: Irradiated human kidney endothelial cells express markers of cellular senescence and SASP factors. Extended data for Fig. 2A. Primary human kidney endothelial cells were induced to undergo senescence by 10 Gy of ionizing radiation (SnC) or not (non-SnC) (n=9 biological replicates) and 20 days later, expression of senescence and SASP markers was measured by qPCR. Means ± SEM, mixed effects model,****p<0.0001.

Figure S5: The SARS-CoV2 spike protein-1 (S1) exacerbates the secretory phenotype of replicative senescent human endothelial cells. Primary human kidney endothelial cells (n=6 biological replicates) were induced to undergo senescence (SnC) with subculturing then treated with 500 ng recombinant S1 or PBS vehicle for 24 hr. SASP-related proteins were measured in the conditioned media (CM) by Luminex xMAP technology, similar to Figure 2A. Mean abundance (pg/mL) of secreted factors that were significantly induced by S1 is illustrated in the heat map and Supplemental Table 3. A mixed effects model was used to test the effect of S1, senescence, and their interaction, taking into account replicate measures for each protein.

Figure S6: SARS-CoV2 spike protein-1 (S1) exacerbates the secretory phenotype of senescent human pre-adipocytes. Analogous to Figure 2A, human adipocyte progenitors isolated from subcutaneous fat biopsies (n=10 subjects) were induced to undergo senescence with 10 Gy of ionizing radiation (SnC) or not (non-SnC). Cells were then treated with 500 ng recombinant S1 for 24 hr. Gene expression was measured by qPCR. Means ± SEM, two-way ANOVA. Arrows and asterisks: grey=vehicle-treated SnC *vs.* non-SnC; black=S1-treated SnC *vs.* non-SnC; red=SnC vehicle *vs.* S1-treated. *p<0.05, **p<0.01, ****p<0.0001.

Figure S7: Expression of viral entry genes in SnC *vs.* non-SnC. A. Primary human lung epithelial cells were treated with recombinant IL-1α (200 μg) for 48 hr or vehicle only. *ACE2* and *TMPRSS2* mRNA were measured by qPCR (n=4 biological replicates). Means ± SEM; mixed effects model. ****p<0.0001. **B.** Primary human kidney endothelial cells were treated with recombinant IL-1α (200 μg) for 48 hr or vehicle only. Gene expression was measured by qPCR 24 hr after treatment and expression is shown relative to vehicle-treated samples. Means ± SEM; mixed effects model; ****p<0.0001. **C.** Human adipocyte progenitors isolated from subcutaneous fat tissue biopsies were induced to undergo senescence by 10 Gy of ionizing radiation (SnC) or not (non-SnC) (n=8 subjects). Gene expression was measured by RNA microarray. Means ± SEM, unpaired Student's *t*-tests, not significant. **D**. Primary human kidney endothelial cells were induced to undergo senescence by 10 Gy of ionizing radiation (SnC) or not (non-SnC) (n=5 biological replicates). *TMPRSS2* mRNA was measured by qPCR and expressed relative to non-senescent kidney endothelial cells. Means ± SEM; unpaired Student's *t*-test. **p<0.01.

Figure S8: NME induces rapid death in old mice of both sexes. A. Extended data for Fig. 3A. Survival curve for 20-24-month-old old male (n=10) and female (n=8) WT mice that were exposed to NME bedding contaminated with murine β-coronavirus MHV for 7 days. **B.** Frequency of tissue resident cells ($CD45^+$, intra-vascular stain negative) in the livers of SPF (n=3 young; n=20 old) and NME (n=8 young; n=17 old) mice. Means ± SEM, two-way ANOVA and *post-hoc* comparison Tukey's Honestly Significant Difference were used to compare the two animal cohorts within a treatment group. ***p<0.001, ****p<0.0001. Arrows and asterisks: grey=SPF old *vs* young; black=NME old *vs* young; red=old SPF *vs* old NME. **C.** Extended data from Fig. 3E. Representative hematoxylin & eosin stain (1) and immunohistochemistry for MHV surface antigen (2) in sections of the intestinal tract of a 20-month-old WT NME mouse. Scale bar=50 μm.

Figure S9: Fisetin improved survival and reduced inflammation in old mice exposed to NME. Extended data from Fig. 4. **A.** Schematic diagram of the survival experiment. Old mice were administered Fisetin 20 mg/kg/day or vehicle only by oral gavage daily x 3 days starting on day 3 following initiation of NME. The 3 days of treatment were repeated every week for 3 weeks (3 days on, 4 days off). Animals were fed standard chow with Fisetin added (500 ppm) *ad libitum* once treatment was initiated. Survival curve for 20-24-month-old male (n=10/group) and female (n=8 vehicle; n=9 Fisetin) WT mice exposed to NME bedding contaminated with the murine β-coronavirus, mouse hepatitis virus (MHV) for 7 days. Log-rank (Mantel Cox test). Separate analyses were run for each sex in order to determine a p value for the effect of Fisetin treatment within a given sex. **B**. A cohort of young (2-month-old) and old (20-month-old) female mice (n=8-10 young + vehicle; 7-10 young + Fisetin; 8-11 old + vehicle; 12-13 old + Fisetin) were exposed to NME as described in Figure 4 for molecular analysis. Tissues were harvested 8-9 days post-

initiation of NME. SASP factor mRNA expression was measured in liver, spleen, kidney, and lung. All expression data were normalized to young mice treated with vehicle. Means ± SEM, two-way ANOVA and *post-hoc* comparison Tukey's Honestly Significant Difference (HSD) used to compare the two animal cohorts within a treatment group. Arrows and asterisks: grey=vehicle-treated young *vs.* old; black=Fisetin-treated young *vs.* old; red=old ± Fisetin. *p<0.05, **p<0.01, ***p<0.001, ****p<0.0001.

Figure S10: Genetic ablation of SnC suppresses inflammation/SASP *in vivo*. A. Extended sex-based survival analysis of NME-exposed old INK-ATTAC mice (n=8 old female + vehicle; n=9 old female + AP20187; n=11 old male + vehicle; n=10 old male + AP20187) from Fig. 5D. Log-rank (Mantel Cox) test. **B.** Extended data for Fig. 5A-D. Young (4 month-old) and old (22-30 month-old) male *INK-ATTAC* mice (n=5 young; n=3-4 old + vehicle; n=4 old + AP20187) were exposed to NME ± AP20187 to ablate SnC as described in Figure 5 and tissues were collected on day 7 post-initiation of NME. Quantification of SASP factor mRNA in multiple tissues. All expression data were expressed relative to vehicle-treated young mice. Means ± SEM. One-way ANOVA. *p<0.05, **p<0.01, ***p<0.001, ****p<0.0001.

Figure S11: Fisetin suppresses mortality and improves adaptive immunity in aged mice challenged with environmental pathogens. Extended data for Fig. 5F-G. **A**. Old (22-26-month-old) mice were treated with Fisetin prior to exposure to NME to determine if senolytics prevent mortality caused by β-coronavirus infection. Mice were pretreated with 20 mg/kg Fisetin (n=19) by oral gavage daily x 2 days or vehicle (n=20). Animals were fed standard chow with Fisetin added (500 ppm) upon initiation of NME. Survival was measured for 45 days post-initiation of

NME. Log-rank (Mantel Cox) test. **B**. Analysis of survival in panel A by sex (n=10/group, except for old female + Fisetin n=9). Log-rank (Mantel Cox) test. Separate analyses were run for each sex to determine a p value for the effect of Fisetin treatment. **C.** Relative MHV antibody score in 22-26-month-old mice in the lifespan cohort on days 16 (n=8) and 21 (n=6) following initiation of NME.

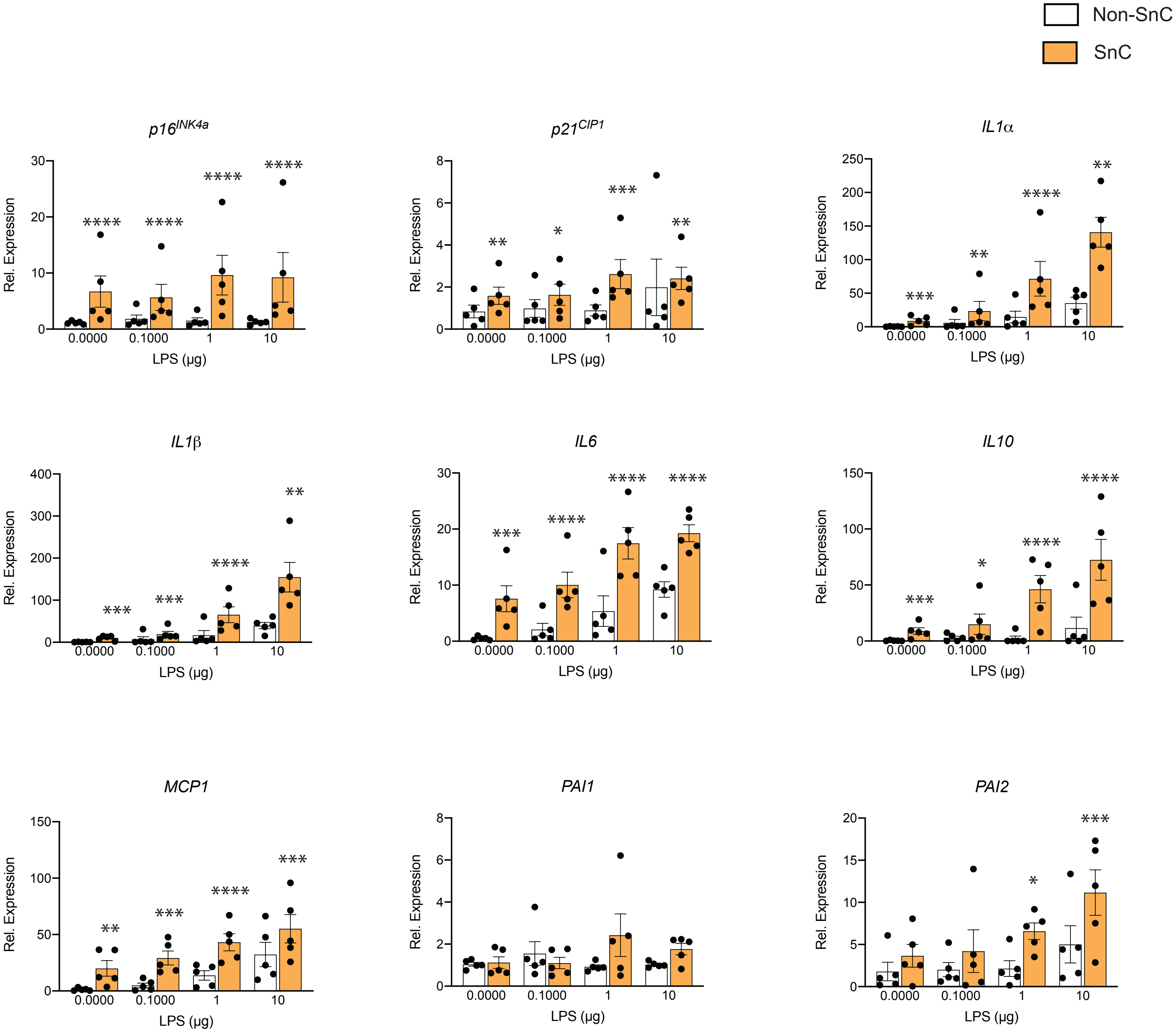

Supplementary Figure 1

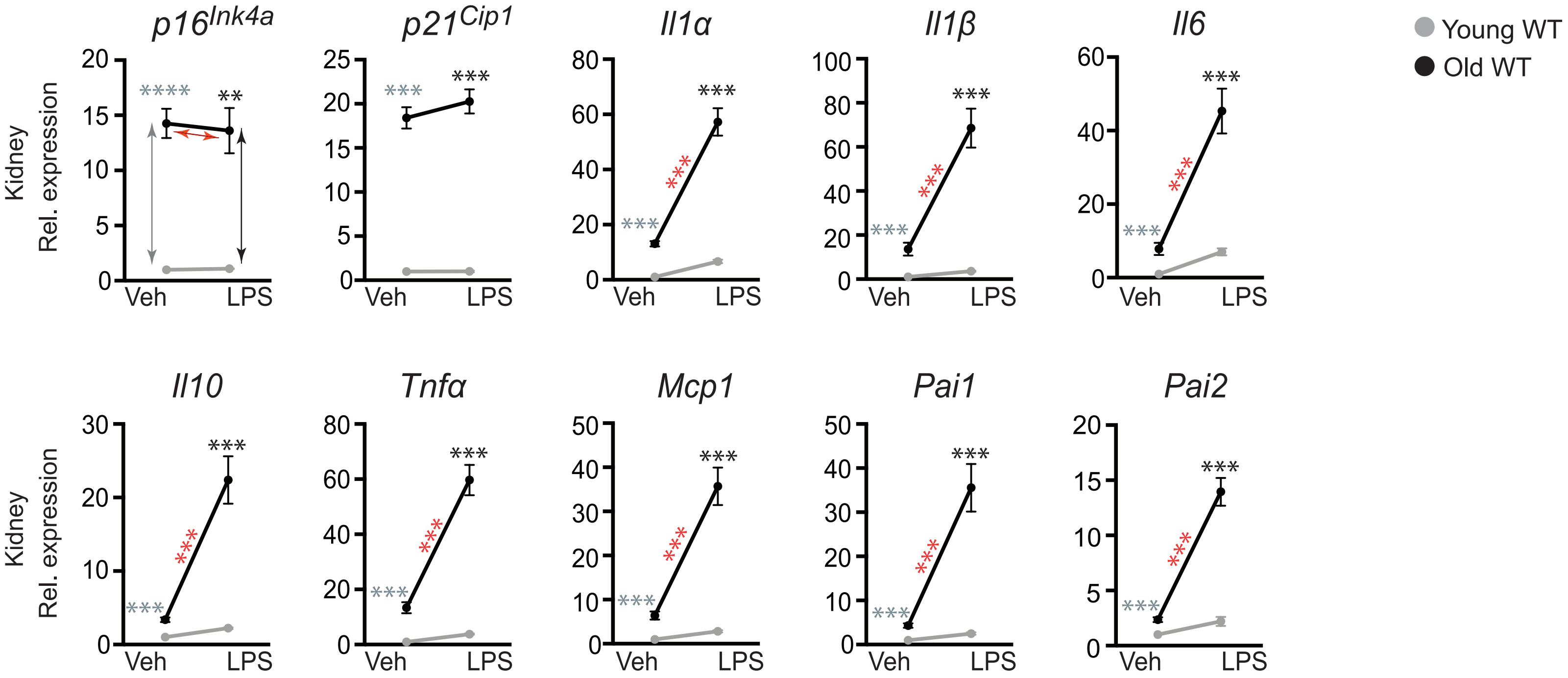

Supplementary Figure 2

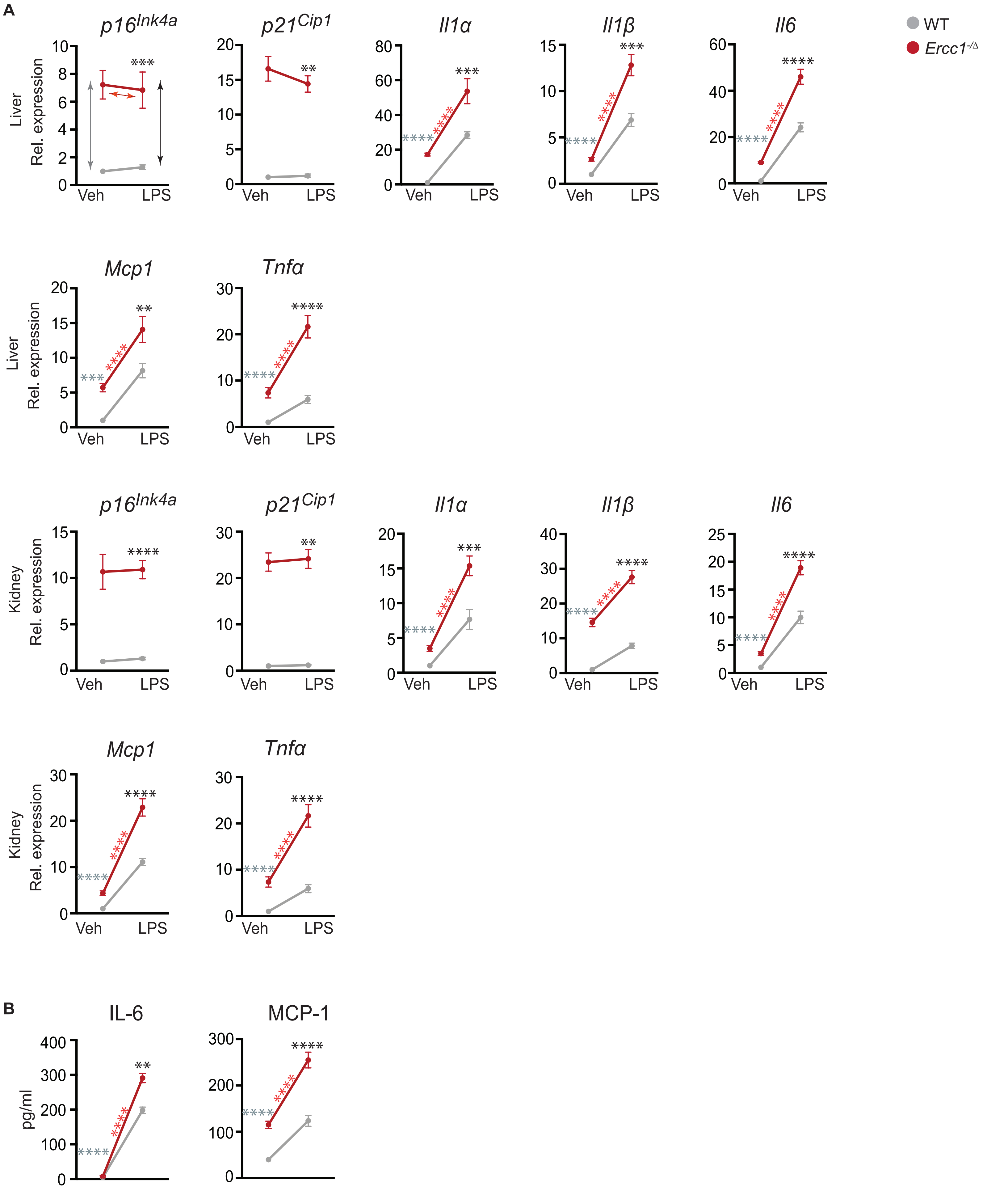

Supplementary Figure 3

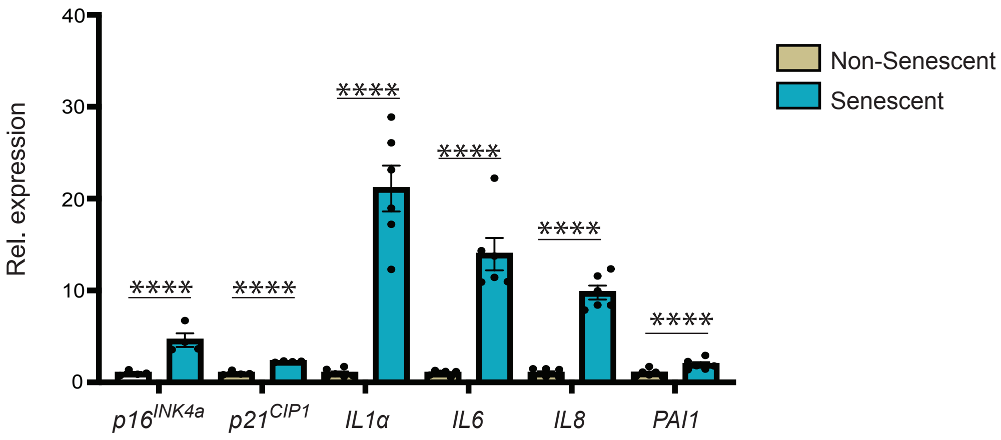

Supplementary Figure 4

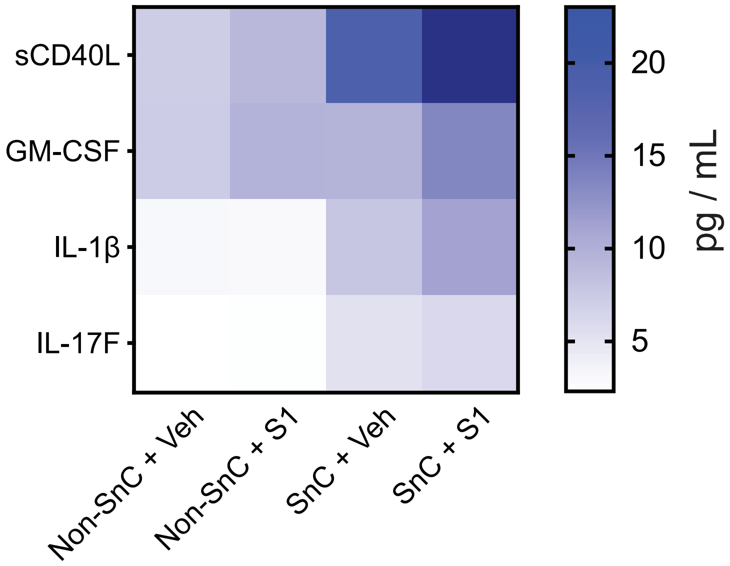

Supplementary Figure 5

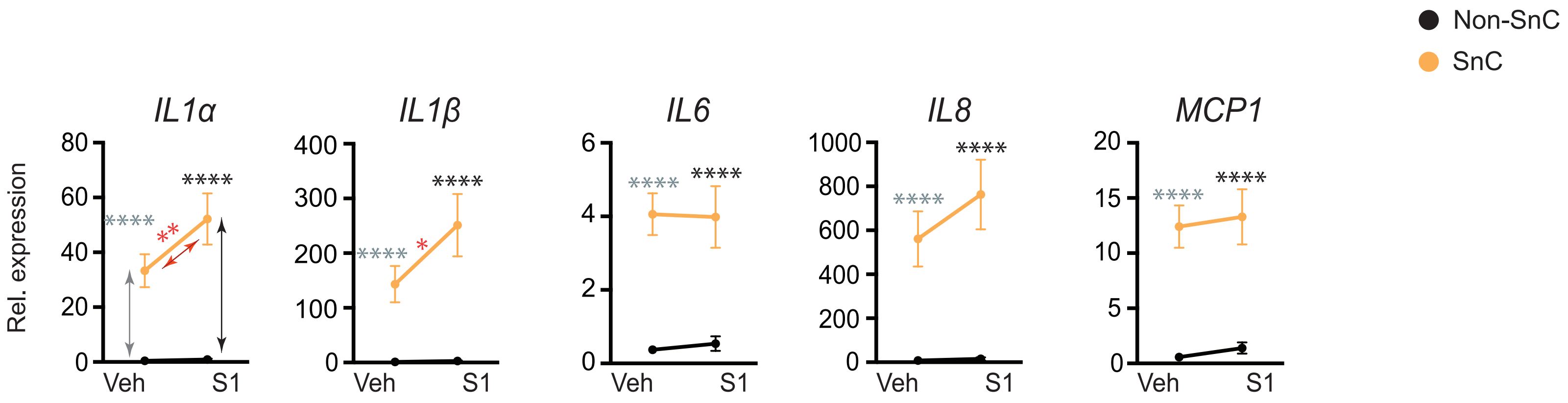

Supplementary Figure 6

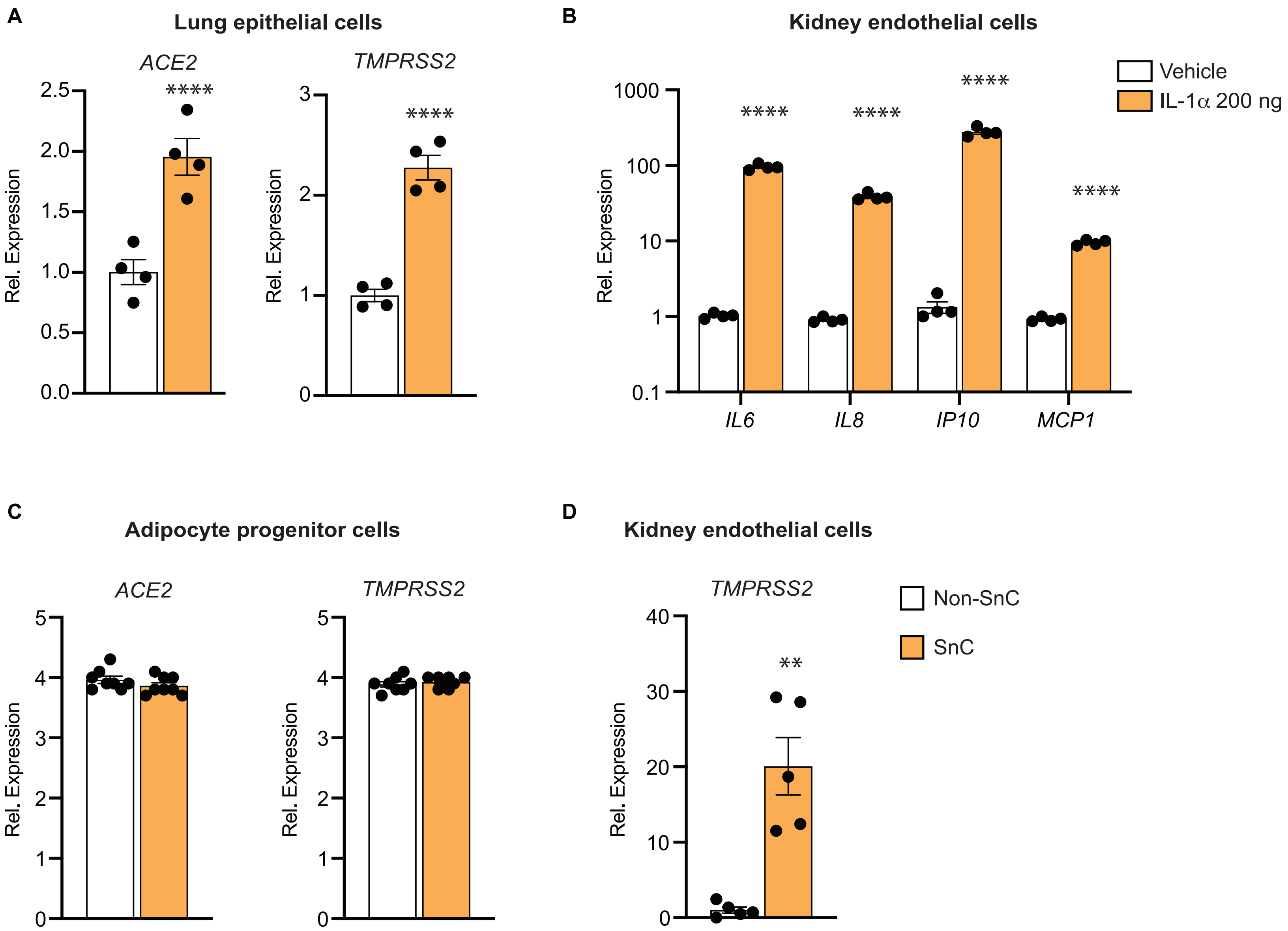

Supplementary Figure 7

A

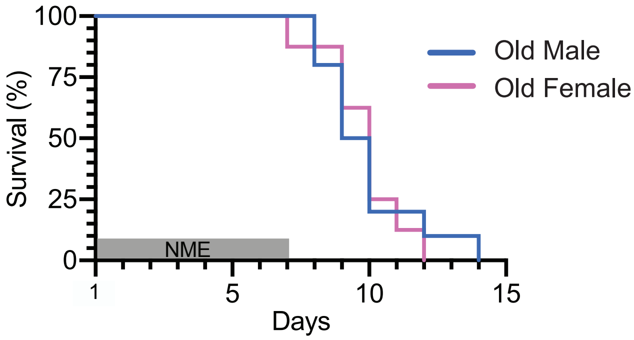

B

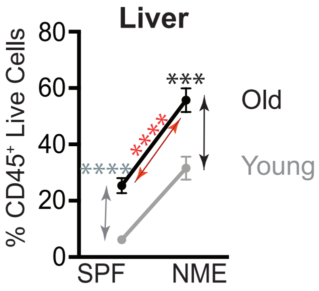

C

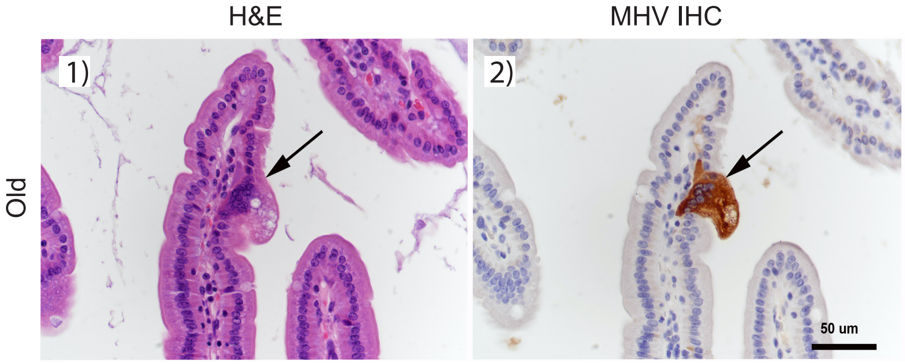

Supplementary Figure 8

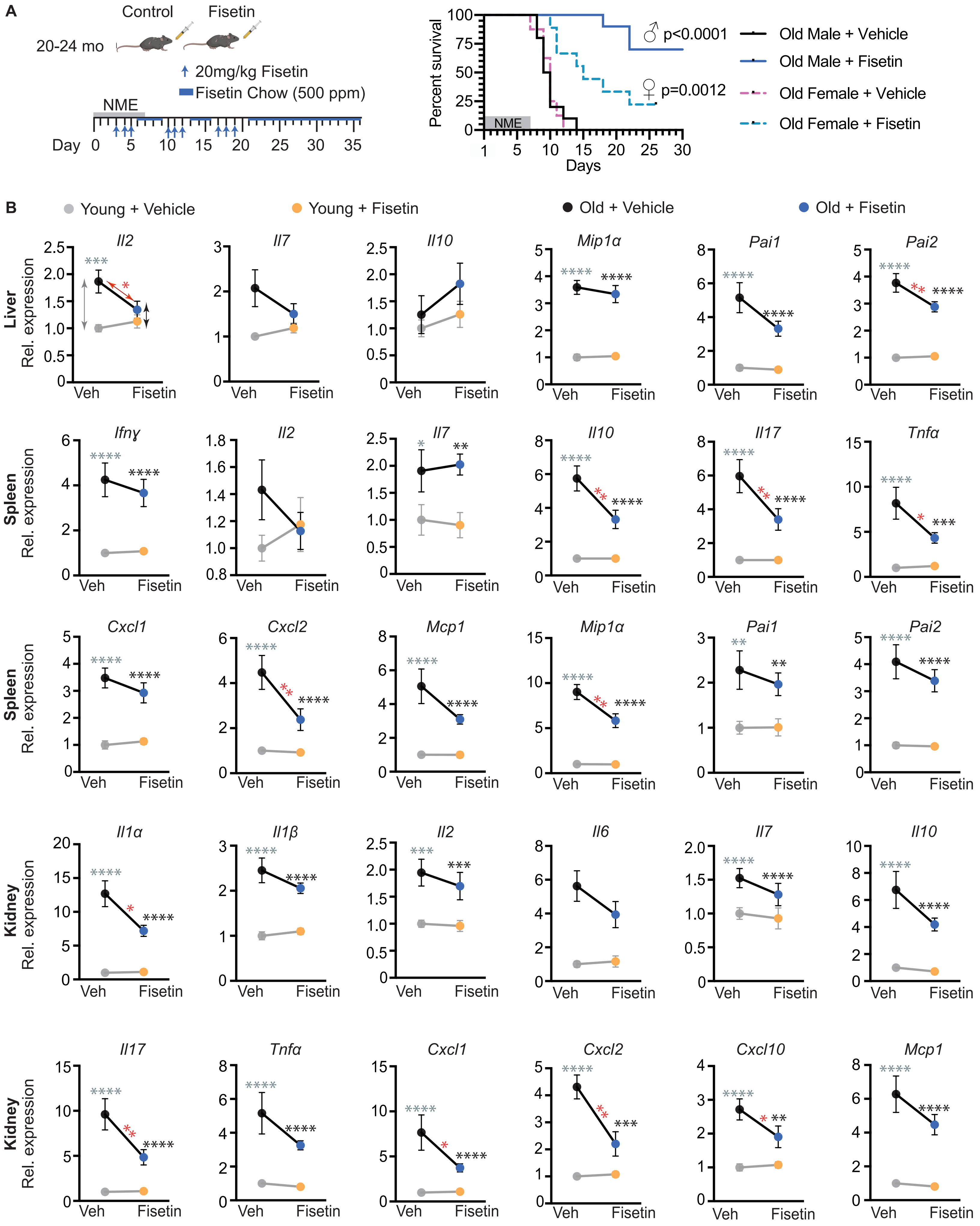

Supplementary Figure 9

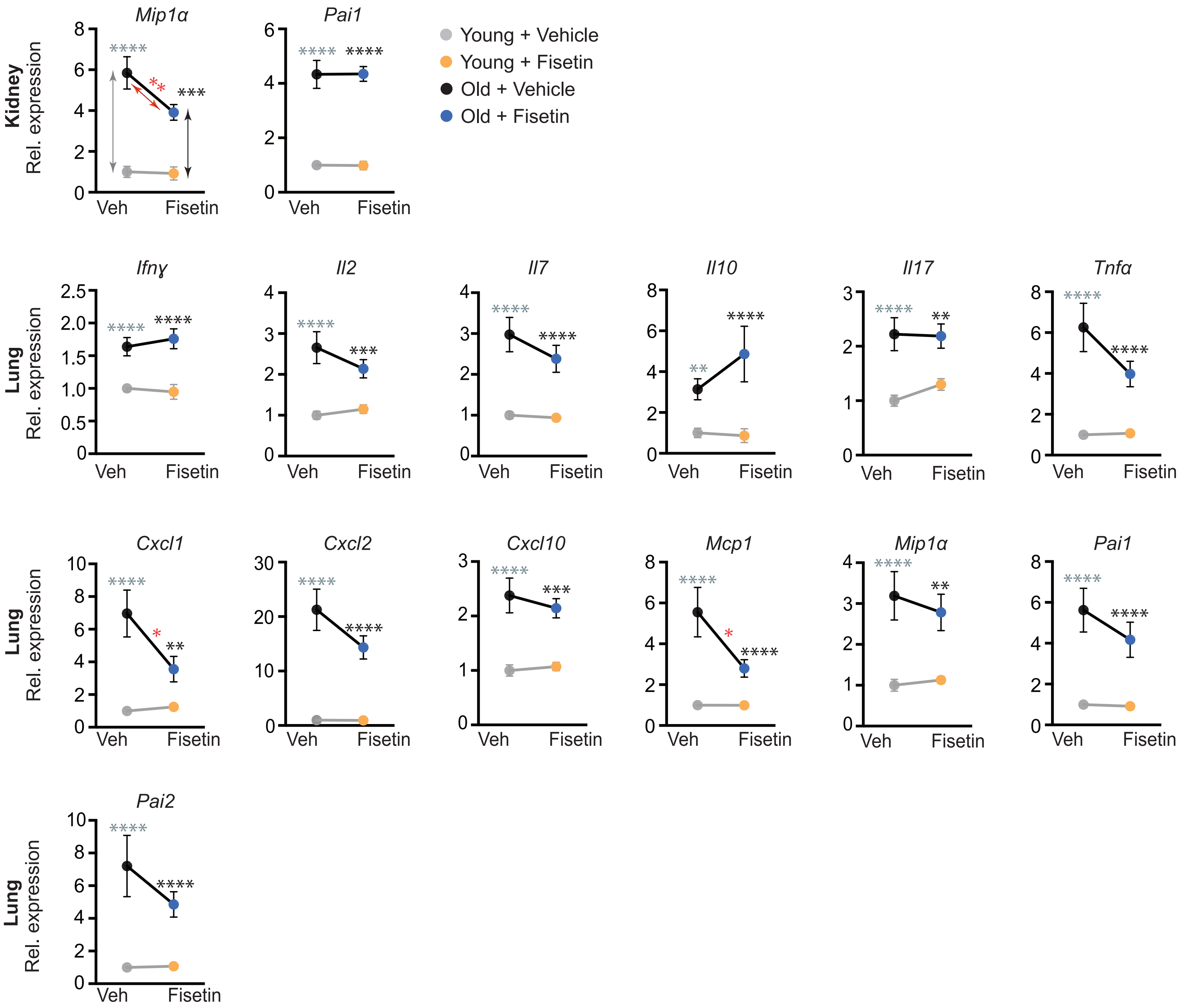

Supplementary Figure 9 (continued)

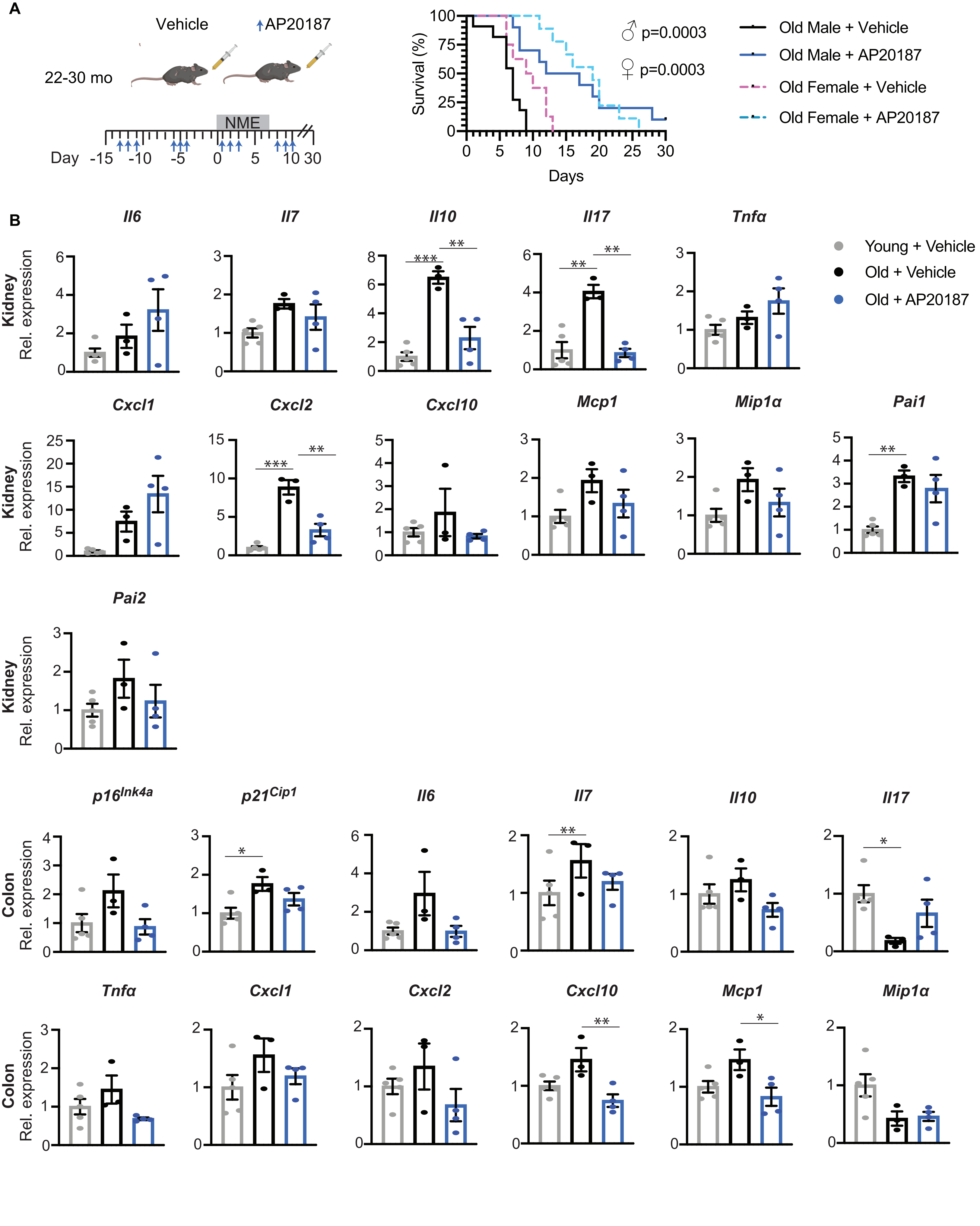

Supplementary Figure 10

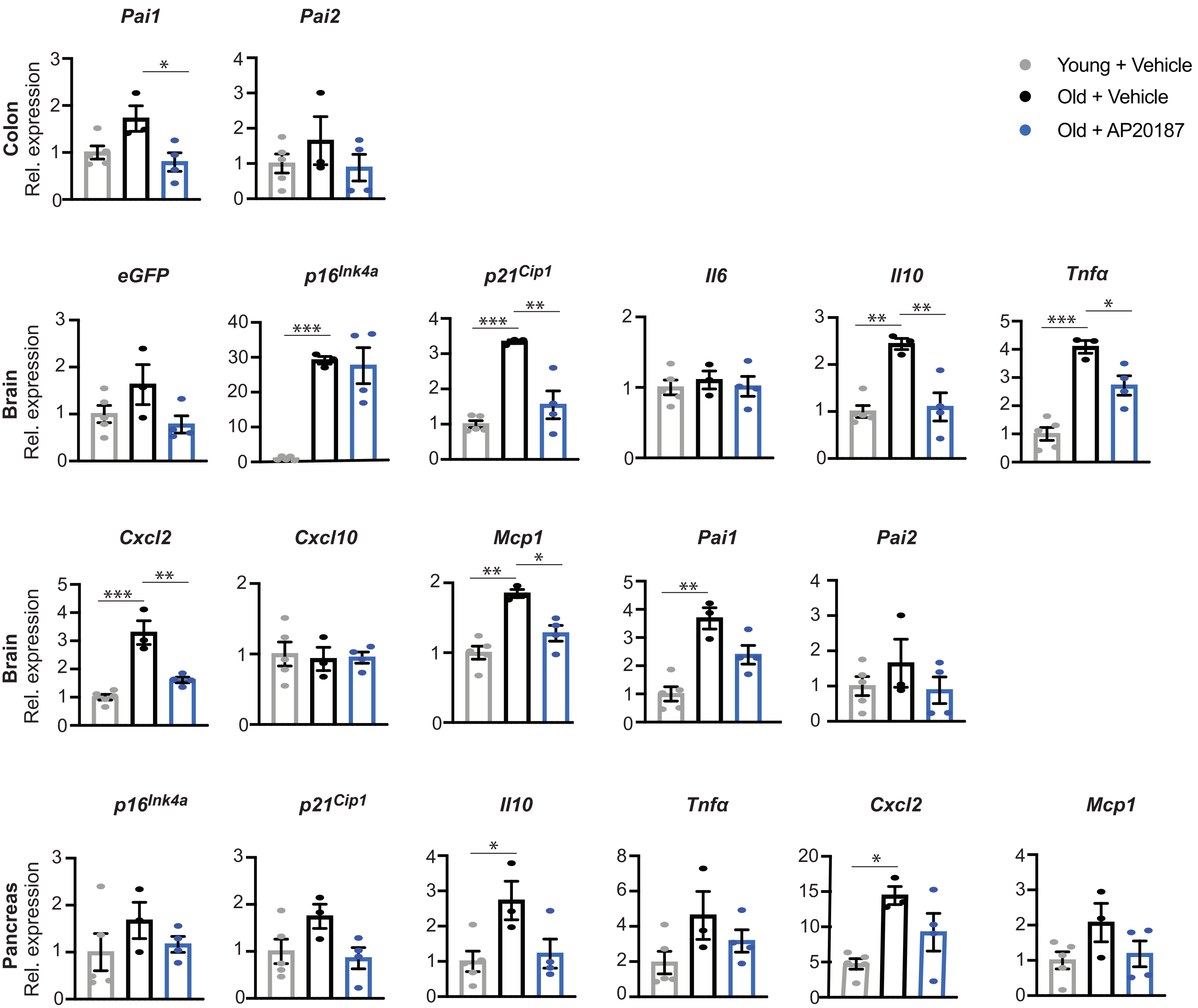

Supplementary Figure 10 (continued)

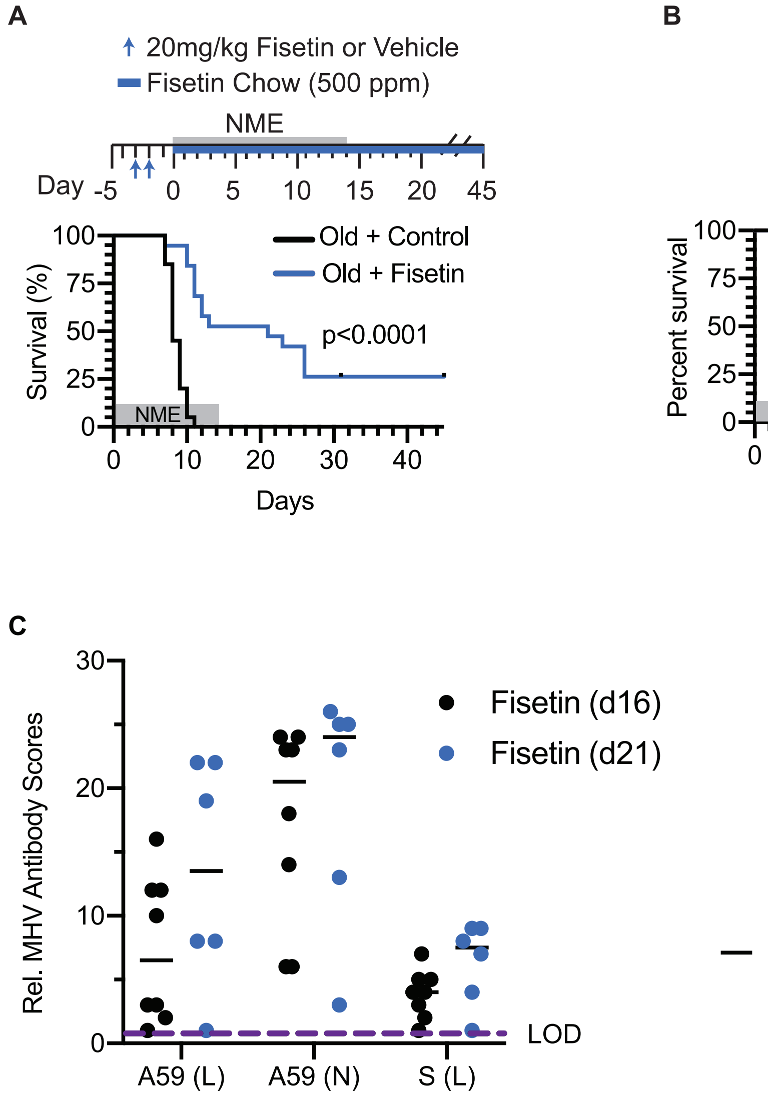

Supplementary Figure 11

Supplemental Table 1. Statistical analysis and p values

Figure 1A

	IL1a	*IL1b*	*IL-6*	*MCP-1*	*IL-10*	*PAI1*	*PAI2*	*p16*	*p21*
Veh vs LPS in SC	<0.0001	<0.0001	<0.0001	<0.0001	<0.0001	0.0227	<0.0001	0.386	0.1049
Veh Non-SC vs SC	<0.0001	<0.0001	<0.0001	0.0138	0.5155	0.2375	0.0679	<0.0001	0.0093
LPS Non-SC vs SC	<0.0001	<0.0001	<0.0001	0.0027	<0.0001	0.0004	<0.0001	<0.0001	0.004
Note: +, zero-skewness log transformed	+	+						+	+

Figure 1B

	Il1a	Il1b	IL-6	MCP-1	IL-10	PAI1	PAI2	p16	p21	Tnf
Veh vs LPS in Old WT	<0.0001	<0.0001	<0.0001	<0.0001	<0.0001	0.0013	0.0001	0.419	0.9885	<0.0001
Veh Young vs Old	<0.0001	<0.0001	<0.0001	<0.0001	<0.0001	<0.0001	<0.0001	<0.0001	<0.0001	<0.0001
LPS Young vs Old	<0.0001	<0.0001	<0.0001	<0.0001	0.0005	<0.0001	<0.0001	0.0064	<0.0001	<0.0001
Note: +, zero-skewness log transformed;++, box cox transformed; +++ rank transformed	+	+	+	+	+	+	+	+++		+

Figure 1C

	IL-6	MCP-1	TNFa
Veh vs LPS in Old WT	<0.0001	<0.0001	<0.0001
Veh Young vs Old	<0.0001	<0.0001	<0.0001
LPS Young vs Old	<0.0001	<0.0001	<0.0001
Note: +, zero-skewness log transformed	+	+	+

Figure 2A

Overall treatment effects in Sc	<0.0001
Overall effects of S1 in Sc vs. Overall effects in non Sc	0.0089

	sCD40L	Eotaxin	Fractalkine	G-CSF	GM-CSF	IFN-a2	IFNy	IL-1a	IL-1b	IL-1RA
Veh vs. S1 in Sc	<0.0001	<0.0001	<0.0001	<0.0001	<0.0001	<0.0001	<0.0001	<0.0001	<0.0001	<0.0001
Effects of S1 in SC vs effects in NonSc	0.0032	0.0094	0.0451	0.5482	0.7491	0.0153	0.1601	0.2668	0.0801	0.0007
Note: +, zero-skewness log transformed;++, box cox transformed; +++ rank transformed	+	+		+	+	+	+	+	+	+

	IL-2	IL-3	Il-5	IL-6	IL-8	IL-10	IL-12p40	IL-12p70	IL-13	IL-17F
Veh vs. S1 in Sc	<0.0001	0.0017	<0.0001	<0.0001	<0.0001	<0.0001	<0.0001	<0.0001	<0.0001	<0.0001
Effects of S1 in SC vs effects in NonSc	0.0418	0.2146	0.0139	0.9095	0.6694	0.0542	0.0105	0.1076	0.0021	0.0069
Note: +, zero-skewness log transformed;++, box cox transformed; +++ rank transformed	+	+++	++		+	+	+	+	+	+

	IL-18	IL-22	IL-27	IP-10	MIG/CXCL9	PDGF-AB/BI	RANTES	TGFa	TNFa	TNFb
Veh vs. S1 in Sc	<0.0001	<0.0001	0.0004	<0.0001	<0.0001	<0.0001	<0.0001	<0.0001	<0.0001	<0.0001
Effects of S1 in SC vs effects in NonSc	0.0618	0.2376	0.0809	0.3385	0.3084	0.7493	0.7852	0.0027	0.4246	0.0954
Note: +, zero-skewness log transformed;++, box cox transformed; +++ rank transformed				+		+	+		+	+

**Figure 2C**	IFITM1	IFITM2	IFITM3
NonSc CM vs. Sc. CM	0.6453	<0.0001	<0.0001
Sc CM vs. Sc CM+ anti-IL-1a	0.7049	0.0816	0.0901
Sc CM vs. Sc CM+ anti-IL-18	0.7638	0.479	0.1046
Sc CM vs. Sc CM+ anti-IL-1a+anti-IL-18+Anti-PAI1	0.2169	0.002	0.0011
Note: +, zero-skewness log transformed	+		

**Figure 2D**	IFITM1	IFITM2	IFITM3
Veh vs. IL1a (200ng)	0.3900	<0.0001	<0.0001
Veh vs. IL1a (1 ug)	0.1234	<0.0001	<0.0001

Note: +, zero-skewness log transformed	+

Figure 2E	ACE2	TMPRSS2
CM of NonSc preadip[ocytes vs. CM of Sc preadipocytes	0.9908	0.7911
CM of NonSc Kidney endothelial cells vs. CM of Sc kidney endothelial cells	<0.0001	0.0014
CM of NonSc lung epithelial cells vs. CM of Sc lung epithelial cells	<0.0001	0.0259

Figure 2F-1	TMPRSS2
NonSc CM vs. Sc. CM	<0.0001
Sc CM vs. Sc CM+ anti-PAI1	0.1199
Sc CM vs. Sc CM+ anti-IL-1a	<0.0001
Sc CM vs. Sc CM+ anti-IL-18	0.1202
Note: +, zero-skewness log transformed	+

Figure 2F-2	TMPRSS2
Veh vs. IL-1a (200ng)	<0.0001
Veh vs. IL-1a (1 ug)	<0.0001

Figure 2H	
CORRELATION OF P16 AND TMPRSS2 with ID controlled	r=0.7233
Regress tmprss again P16 with ID controlled	R-squared=0.65
	Adj R-squared=0.55

Figure 3B	Liver	Lung	Kidney	Liver	Lung	Kidney
	p16	*p16*	*p16*	*p21*	*p21*	*p21*
SPF vs NME in Old	0.0272	0.1743	0.6427	0.0006	0.0148	0.1081
SPF Young vs Old	0.0028	0.2695	<0.0001	0.7542	0.4048	0.0021
NME Young vs Old	0.0034	0.0327	<0.0001	0.0015	0.1229	0.0146
Note: +, zero-skewness log transformed	+	+	+	+	+	+

	Liver	Lung	Kidney	Liver	Lung	Kidney	Liver	Lung	Kidney
	Il6	*Il6*	*Il6*	*Mcp1*	*Mcp1*	*Mcp1*	*Tnf*	*Tnf*	*Tnf*
SPF vs NME in Old	0.0013	0.2113	0.0003	0.0009	0.0057	0.0001	0.0042	0.1611	0.0002
SPF Young vs Old	0.2609	0.2288	0.0011	0.1167	0.0391	0.1184	0.1312	0.2166	0.1843
NME Young vs Old	0.0336	0.5996	0.0001	0.5117	0.1229	0.0132	0.6855	0.8083	0.0017
Note: +, zero-skewness log transformed	+	+	+	+	+	+	+	+	

Figure 3C				
	IL-6	IL-10	CCL11	TNF
SPF vs NME in Old	0.0024	0.0872	<0.0001	0.0056
SPF Young vs Old	0.3077	0.7975	0.9316	0.8639
NME Young vs Old	0.0030	0.2706	0.0002	0.0034
Note: +, zero-skewness log transformed;++, box cox transformed; +++ rank transformed	++	++		++

Figure 4D	
	MHV
Veh vs Fisetin in Old	0.1627
Fisetin Young vs Old	<0.0001
Veh Young vs Old	<0.0001
Note: +, zero-skewness log transformed;	+

Figure 4E

	Liver	Kidney	Lung	Spleen	Liver	Kidney	Lung	Spleen
	p16	*p16*	*p16*	*p16*	*p21*	*p21*	*p21*	*p21*
Veh vs Fisetin in Old	0.0023	0.0846	0.0345	0.7789	0.0085	<0.0001	0.2120	0.0198
Fisetin Young vs Old	<0.0001	<0.0001	<0.0001	<0.0001	0.0008	<0.0001	<0.0001	<0.0001
Veh Young vs Old	<0.0001	<0.0001	<0.0001	<0.0001	<0.0001	<0.0001	<0.0001	<0.0001
Note: +, zero-skewness log transformed;++, box cox transformed; +++ rank transformed		+++	+++	+++	+		+	+

Figure 4F

	Ifnr	*Il1a*	*Il1b*	*Il6*	*Il17*	*Tnfa*	*Cxcl1*	*Cxcl2*	*Cxcl10*	*Mcp1*
Veh vs Fisetin in Old	0.0149	0.0004	0.0058	0.1128	0.0038	0.0087	0.0139	0.0327	0.0018	0.0066
Fisetin Young vs Old	<0.0001	<0.0001	0.0005	0.1602	0.0005	0.0002	0.0002	<0.0001	<0.0001	0.0110
Veh Young vs Old	<0.0001	<0.0001	<0.0001	0.0001	<0.0001	<0.0001	<0.0001	<0.0001	<0.0001	<0.0001
Note: +, zero-skewness log transformed;++, box cox transformed; +++ rank transformed	+	+	+	+	+	+	+	+++		

Figure 4G

	IL-1b	IL-6	MCP-1	TNFa
Veh vs Fisetin in Old	0.0027	0.0273	0.0125	0.0012
Fisetin Young vs Old	<0.0001	<0.0001	<0.0001	<0.0001
Veh Young vs Old	<0.0001	<0.0001	<0.0001	<0.0001
Note: +, zero-skewness log transformed;++, box cox transformed; +++ rank transformed	+	+++	+++	

**Figure 5B**	MHV
Young Veh vs Old Veh	0.005
Old Veh vs Old SP20187	0.048

**Figure 5B**	eGFP	p16	p21
Young Veh vs Old Veh	<0.001	0.004	0.001
Old Veh vs Old SP20187	<0.001	0.162	0.03

**Supplemental Figure 1**	*IL1a*	*IL1b*	*IL-6*	*MCP-1*	*IL-10*	*PAI1*	*PAI2*	*p16*	*p21*
Non-SC vs SC 0 ug LPS	0.0004	0.0001	0.0002	0.0041	0.0002	0.7391	0.2947	<0.0001	0.007
Non-SC vs SC 0.1 ug LPS	0.002	0.0009	<0.0001	0.0002	0.0402	0.3076	0.2175	<0.0001	0.0396
Non-SC vs SC 1 ug LPS	<0.0001	<0.0001	<0.0001	<0.0001	<0.0001	0.1551	0.0128	<0.0001	0.0001
Non-SC vs SC 10 ug LPS	0.0011	0.0017	<0.0001	0.0005	<0.0001	0.1726	0.0005	<0.0001	0.0029
Note: +, zero-skewness log transformed;++, box cox transformed; +++ rank transformed	+	+++			+	+		+	+

**Supplemental Figure 2**	*Il1a*	*Il1b*	*Il6*	*Il10*	*Mcp1*	*Pai1*	*Pai2*	*Tnf*	*p16*	*p21*
Veh vs LPS in Old WT	<0.001	0.0001	<0.001	<0.001	<0.001	0.0001	0.0001	<0.001	0.6998	0.3617
Veh Young vs Old	<0.001	<0.001	<0.001	<0.001	<0.001	<0.001	<0.001	<0.001	<0.001	<0.001
LPS Young vs Old	<0.001	<0.001	<0.001	<0.001	<0.001	<0.001	<0.001	<0.001	0.0029	0.0003
Note: +, zero-skewness log transformed	+	+	+	+	+	+	+	+	+++	+++

**Supplemental Figure 3A Liver**	*p16*	*p21*	*Il1a*	*Il1b*	*Il6*	*Mcp1*	*Tnf*
Veh vs LPS in Ercc1-/Δ	0.7497	0.5939	<0.0001	<0.0001	<0.0001	0.0002	<0.0001
Veh WT vs Ercc1-/Δ	0.8699	1	<0.0001	<0.0001	<0.0001	<0.0001	<0.0001
LPS WT vs Ercc1-/Δ	0.0004	0.0024	0.0005	0.0001	<0.0001	0.0027	<0.0001
Note: +, zero-skewness log transformed;++, box cox transformed; +++ rank transformed		+++					

**Supplemental Figure 3A Kidney**	*p16*	*p21*	*Il1a*	*Il1b*	*Il6*	*Mcp1*	*Tnf*
Veh vs LPS in Ercc1 - A versus B	0.8771	0.5881	<0.0001	<0.0001	<0.0001	<0.0001	<0.0001

A vs. C	0.8011	0.3447	<0.0001	<0.0001	<0.0001	<0.0001	<0.0001
B vs. D	<0.0001	0.001	0.0002	<0.0001	<0.0001	<0.0001	<0.0001
Note: +, zero-skewness log transformed;++, box cox transformed; +++ rank transformed		+++					

**Supplemental Figure 3B Serum**	IL6	MCP1
Veh vs LPS in Ercc1 - A versus B	<0.0001	<0.0001
LPS effect in Ercc1 vs effect in WT - Slope of line 1 versus 2	0.4245	0.0282
A vs. C	<0.0001	<0.0001
B vs. D	0.0089	<0.0001
Note: +, zero-skewness log transformed;++, box cox transformed; +++ rank transformed	+++	

**Supplemental Figure 4**	p16	p21	IL-1α	IL-1β	IL-6	IL-8	PAI-1
Non-SC vs. SC	<0.0001	<0.0001	<0.0001	<0.0001	<0.0001	<0.0001	<0.0001
Note: +, zero-skewness log transformed			+	+	+		

**Supplemental Figure 5**	sCD40L	GM-CSF	IL-1β	IL-17F
Veh vs S1 in Scns	0.0357	0.0576	0.008	0.0185
S1 effect inScns vs effect in NonScns	0.3461	0.5992	0.041	0.3755
Veh vs S1 in Non Scns	0.0381	0.0314	0.0617	0.0132
Scns Veh vs. Non Scns Veh	<0.0001	0.0342	<0.0001	<0.0001
Note: +, zero-skewness log transformed;++, box cox transformed; +++ rank transformed				+++

**Supplemental Figure 6**	*IL1a*	*IL1b*	*IL-6*	*IL-8*	*MCP-1*
Veh vs S1 in Sc - A versus B	0.0034	0.046	0.5589	0.0865	0.709
Veh Non-SC vs SC	<0.0001	<0.0001	<0.0001	<0.0001	<0.0001
S1 Non-SC vs SC	<0.0001	<0.0001	<0.0001	<0.0001	<0.0001
Note: +, zero-skewness log transformed	+	+	+		+

**Supplemental Figure 7A**	ACE2	TMPRSS2
Veh vs. IL-1a (200ng)	<0.0001	<0.0001

**Supplemental Figure 7B**	IL-6	IL-8	IP10	MCP1
Vehicle vs IL-1a tx	<0.0001	<0.0001	<0.0001	<0.0001

**Supplemental Figure 7C**	ACE2	TMPRSS2
Non-SC vs. SC	0.2342	0.4994

**Supplemental Figure 7D**	TMPRSS2
Non-SC vs. SC	0.0011

**Supplemental Figure 8B**	CD45+ cells
SPF vs NME in Old	<0.0001
SPF Young vs Old	<0.0001
NME Young vs Old	0.0001

**Supplemental Figure 9B**	Liver	Liver	Liver	Liver	Liver	Liver
	Il2	*Il7*	*Il10*	*Mip1a*	*Pai1*	*Pai2*
Veh vs Fisetin in Old	0.0193	0.3649	0.2438	0.6787	0.0598	0.0033

Fisetin Young vs Old	0.3850	0.6882	0.4845	<0.0001	<0.0001	<0.0001
Veh Young vs Old	0.0006	0.0726	0.8230	<0.0001	<0.0001	<0.0001
Note: +, zero-skewness log transformed;++, box cox transformed; +++ rank transformed	+	+	+	+++	+	

	Spleen	Spleen	Spleen	Spleen	Spleen	Spleen
	Ifng	*Il2*	*Il7*	*Il10*	*Il17*	*Tnfa*
Veh vs Fisetin in Old	0.5773	0.2232	0.6524	0.0055	0.0049	0.0279
Fisetin Young vs Old	<0.0001	0.8516	0.0047	<0.0001	<0.0001	0.0007
Veh Young vs Old	<0.0001	0.1100	0.0378	<0.0001	<0.0001	<0.0001
Note: +, zero-skewness log transformed;++, box cox transformed; +++ rank transformed	+		+	+++	+	+

	Spleen	Spleen	Spleen	Spleen	Spleen	Spleen
	Cxcl1	*Cxcl2*	*Mcp1*	*Mip1a*	*Pai1*	*Pai2*
Veh vs Fisetin in Old	0.1815	0.0027	0.0669	0.0069	0.5973	0.2660
Fisetin Young vs Old	<0.0001	<0.0001	<0.0001	<0.0001	0.0041	<0.0001
Veh Young vs Old	<0.0001	<0.0001	<0.0001	<0.0001	0.0028	<0.0001
Note: +, zero-skewness log transformed;++, box cox transformed; +++ rank transformed	+	+	+	+++	+	+

	Kidney	Kidney	Kidney	Kidney	Kidney	Kidney
	Il1a	*Il1b*	*Il2*	*Il6*	*Il7*	*Il10*
Veh vs Fisetin in Old	0.0173	0.0628	0.3457	0.0573	0.2116	0.0950
Fisetin Young vs Old	<0.0001	<0.0001	0.0009	<0.0001	0.0535	<0.0001
Veh Young vs Old	<0.0001	<0.0001	0.0003	<0.0001	0.0138	<0.0001
Note: +, zero-skewness log transformed;++, box cox transformed; +++ rank transformed	+		+	+	+	+

	Kidney	Kidney	Kidney	Kidney	Kidney	Kidney
	Il17	*Tnfa*	*Cxcl1*	*Cxcl2*	*Cxcl10*	*Mcp1*
Veh vs Fisetin in Old	0.0070	0.0740	0.0308	0.0018	0.0357	0.1547
Fisetin Young vs Old	<0.0001	<0.0001	<0.0001	0.0005	0.0039	<0.0001
Veh Young vs Old	<0.0001	<0.0001	<0.0001	<0.0001	<0.0001	<0.0001
Note: +, zero-skewness log transformed;++, box cox transformed; +++ rank transformed	+++	+	+	+	+	+++

	Kidney	Kidney
	Mip1a	*Pai1*
Veh vs Fisetin in Old	0.0084	0.8260
Fisetin Young vs Old	0.0002	<0.0001
Veh Young vs Old	<0.0001	<0.0001
Note: +, zero-skewness log transformed;++, box cox transformed; +++ rank transformed		+++

	Lung	Lung	Lung	Lung	Lung	Lung
	Ifng	*Il2*	*Il7*	*Il10*	*Il7*	*Tnfa*
Veh vs Fisetin in Old	0.4869	0.2290	0.2332	0.4918	0.9646	0.0521
Fisetin Young vs Old	0.0001	0.0004	<0.0001	<0.0001	0.0025	<0.0001
Veh Young vs Old	0.0011	<0.0001	<0.0001	0.0029	<0.0001	<0.0001
Note: +, zero-skewness log transformed;++, box cox transformed; +++ rank transformed		+	+	+	+	+

	Lung	Lung	Lung	Lung	Lung	Lung
	Cxcl1	*Cxcl2*	*Cxcl10*	*Mcp1*	*Mip1a*	*Pai1*
Veh vs Fisetin in Old	0.0189	0.1465	0.3793	0.0199	0.6119	0.2253
Fisetin Young vs Old	0.0011	<0.0001	0.0003	<0.0001	0.0024	<0.0001
Veh Young vs Old	<0.0001	<0.0001	<0.0001	<0.0001	0.0001	<0.0001
Note: +, zero-skewness log transformed;++, box cox transformed; +++ rank transformed	+	+		+	+	+

	Lung

	Pai2
Veh vs Fisetin in Old	0.2809
Fisetin Young vs Old	<0.0001
Veh Young vs Old	<0.0001
Note: +, zero-skewness log transformed;++, box cox transformed; +++ rank transformed	+

Figure 10B

Kidney	*Il10*	*Il17*	*Cxcl2*	*Pai1*
Young Veh vs Old Veh	<0.001	0.001	<0.001	0.005
Old Veh vs Old SP20187	0.001	0.001	0.001	0.633

Colon	*p21*	*Il7*	*Il17*	*Cxcl10*	*Mcp1*	*Pai1*
Young Veh vs Old Veh	0.024	0.002	0.028	0.065	0.105	0.068
Old Veh vs Old SP20187	0.287	0.224	0.233	0.009	0.034	0.027

Brain	*p16*	*p21*	*Il10*	*Tnfa*	*Cxcl2*	*Mcp1*	*Pai1*
Young Veh vs Old Veh	<0.001	<0.001	0.002	<0.001	<0.001	0.001	0.001
Old Veh vs Old SP20187	0.941	0.002	0.005	0.026	0.001	0.011	0.056

Pancreas	*Il10*	*Cxcl2*
Young Veh vs Old Veh	0.035	0.01
Old Veh vs Old SP20187	0.076	0.174

Supplemental Table 2. Primary human kidney endothelial cells (n=9 biological replicates) were induced to undergo senescence with 10 Gy of ionizing radiation (SnC) or not (non-SnC) then treated with 500 ng recombinant S1 or PBS vehicle for 24 hr. Thirty SASP-related proteins were measured in the conditioned media (CM) by Luminex xMAP technology. Relative abundance induced by S1 (normalized to vehicle treated non-senescent cells (non-SnC + Veh)) is illustrated in the heat map. A mixed effects model was used to test the effect of S1, senescence, and their interaction, taking into account replicate measures within a subject for each protein as well as the composite score. Margin effects of SnC in the treatment group also were tested under the mixed effects model framework. Overall, the effect of S1 on SnC was significantly more pronounced than on non-SnC (composite score change $p<0.0089$). Note: ns not significant; + zero-skewedness log transformed; ++ box cox transformed; +++ rank transformed.

	Non-SnC + Veh	Non-SnC + S1	SnC + Veh	SnC + S1	Veh vs. S1 in SnC	Overall treatment effects in SnC	Effects of S1 in SnC vs effects in Non-SnC	Overall effects of S1 in SnC vs. Overall effects in Non-SnC	Note
**sCD40L**	5.04 ± 1.15	4.00 ± 0.65	5.97 ± 1.05	14.00 ± 4.89	<0.0001		0.0032		+
**Fractalkine**	16.24 ± 2.59	12.12 ± 1.77	40.15 ± 9.07	60.51 ± 14.64	<0.0001		0.0094		+
**Eotaxin**	4.02 ± 0.54	3.44 ± 0.37	5.04 ± 0.67	7.67 ± 2.02	<0.0001		0.0451		
**G-CSF**	36.55 ± 5.23	42.04 ± 8.45	252.07 ± 51.92	420.10 ± 131.35	<0.0001		0.5482		+
**GM-CSF**	22.80 ± 4.79	23.86 ± 4.09	246.54 ± 64.34	438.06 ± 147.85	<0.0001		0.7491		+
**IFN-α2**	3.62 ± 0.58	2.79 ± 0.33	4.37 ± 0.55	10.66 ± 4.01	<0.0001		0.0153		+
**IFNγ**	0.29 ± 0.06	0.27 ± 0.06	0.69 ± 0.07	0.90 ± 0.17	<0.0001		0.1601		+
**IL-1α**	0.70 ± 0.10	0.64 ± 0.10	10.19 ± 1.70	16.04 ± 4.14	<0.0001		0.2668		+
**IL-1β**	2.20 ± 0.44	1.86 ± 0.22	3.08 ± 0.40	7.05 ± 2.55	<0.0001		0.0801		+
**IL-1RA**	0.41 ± 0.05	0.32 ± 0.02	0.46 ± 0.04	0.96 ± 0.30	<0.0001		0.0007		+
**IL-2**	0.05 ± 0.01	0.03 ± 0.01	0.16 ± 0.08	0.56 ± 0.20	<0.0001		0.0418		+
**IL-3**	0.11 ± 0.06	0.05 ± 0.02	0.19 ± 0.03	0.76 ± 0.37	0.0017		0.2146		+++
**IL-5**	0.07 ± 0.01	0.06 ± 0.01	0.08 ± 0.01	0.21 ± 0.09	<0.0001		0.0139		++
**IL-6**	413.94 ± 146.76	458.96 ± 165.30	1645.24 ± 73.89	1663.38 ± 93.12	<0.0001		0.9095		
**IL-8**	2140.10 ± 493.14	2167.84 ± 548.41	5041.39 ± 1086.39	5786.21 ± 1581.28	<0.0001	**<0.0001**	0.6694	**0.0089**	+
**IL-10**	0.29 ± 0.07	0.22 ± 0.04	0.55 ± 0.11	1.10 ± 0.38	<0.0001		0.0542		+
**IL-12p40**	5.17 ± 0.74	4.49 ± 0.85	7.38 ± 0.71	15.45 ± 4.69	<0.0001		0.0105		+
**IL-12p70**	0.31 ± 0.10	0.22 ± 0.04	0.43 ± 0.10	1.49 ± 0.80	<0.0001		0.1076		+
**IL-13**	3.49 ± 0.97	1.28 ± 0.58	5.10 ± 0.81	12.39 ± 5.47	<0.0001		0.0021		+
**IL-17F**	0.84 ± 0.27	0.91 ± 0.21	0.95 ± 0.25	2.14 ± 0.90	<0.0001		0.0069		+
**IL-18**	0.16 ± 0.02	0.13 ± 0.01	0.31 ± 0.07	0.49 ± 0.12	<0.0001		0.0618		
**IL-22**	1.02 ± 1.02	0.00 ± 0.00	22.51 ± 5.03	32.96 ± 5.73	<0.0001		0.2376		
**IL-27**	43.40 ± 6.93	42.72 ± 6.40	52.19 ± 11.17	71.38 ± 15.52	0.0004		0.0809		
**IP-10**	2.73 ± 0.57	3.93 ± 0.97	22.35 ± 9.48	33.01 ± 11.93	<0.0001		0.3385		+
**MIG/CXCL9**	0.67 ± 0.10	0.52 ± 0.08	3.73 ± 1.10	5.01 ± 1.45	<0.0001		0.3084		
**PDGF-AB/BB**	386.58 ± 128.88	385.43 ± 131.82	1577.39 ± 154.89	1608.97 ± 171.04	<0.0001		0.7493		+
**RANTES**	62.98 ± 4.15	68.21 ± 5.85	262.88 ± 36.28	316.34 ± 67.26	<0.0001		0.7852		+
**TGFα**	1.40 ± 0.27	0.82 ± 0.19	2.85 ± 0.37	3.69 ± 0.59	<0.0001		0.0027		
**TNFα**	16.09 ± 2.86	15.93 ± 3.05	30.14 ± 6.06	37.64 ± 8.78	<0.0001		0.4246		+
**TNFβ**	2.60 ± 0.46	2.34 ± 0.42	6.15 ± 0.52	7.63 ± 0.86	<0.0001		0.0954		+
**EGF**	5724.33 ± 1052.49	4901.56 ± 676.40	5566.82 ± 1091.56	6152.77 ± 1428.78	ns		ns		
**FGF-2**	1122.03 ± 64.12	1129.72 ± 52.46	1353.99 ± 53.11	1428.08 ± 76.02	ns		ns		
**FLT-3L**	1.53 ± 0.46	1.16 ± 0.24	0.90 ± 0.14	1.15 ± 0.24	ns		ns		
**GROα**	1142.25 ± 139.30	1183.24 ± 126.26	1833.71 ± 299.99	2203.46 ± 474.36	ns		ns		
**IL-4**	0.12 ± 0.03	0.11 ± 0.03	0.12 ± 0.03	0.17 ± 0.04	ns		ns		
**IL-9**	1.79 ± 0.39	1.68 ± 0.36	2.44 ± 0.38	2.98 ± 0.57	ns	ns	ns	ns	
**IL-15**	5.73 ± 1.94	5.61 ± 1.85	6.24 ± 1.40	7.39 ± 1.57	ns		ns		
**MCP-1**	1156.74 ± 128.61	877.49 ± 64.76	2111.26 ± 335.86	2288.87 ± 410.78	ns		ns		
**MCP-3**	85.21 ± 2.27	88.68 ± 3.43	131.66 ± 37.11	126.68 ± 35.54	ns		ns		
**M-CSF**	132.45 ± 31.13	116.47 ± 24.23	205.77 ± 46.35	218.39 ± 49.77	ns		ns		
**PDGF-AA**	795.06 ± 225.10	777.66 ± 211.66	434.76 ± 57.33	416.70 ± 54.90	ns		ns		

Supplemental Table 3. Primary human kidney endothelial cells (n=6 biological replicates) were induced to undergo senescence (SnC) with subculturing then treated with 500 ng recombinant S1 or PBS vehicle for 24 hr. Thirty SASP-related proteins were measured in the conditioned media (CM) by Luminex xMAP technology. Mean abundance (pg/mL) of secreted factors that were significantly induced by S1 is illustrated in the heat map. A mixed effects model was used to test the effect of S1, senescence, and their interaction, taking into account replicate measures for each protein. Note: + zero-skewedness log transformed.

	Non-SnC + Veh	Non-SnC + S1	SnC + Veh	SnC + S1	Veh vs. S1 in SnC
**sCD40L**	7.34167 ± 0.3852	9.25667 ± 1.7731	18.6833 ± 1.83973	23.5417 ± 1.61923	0.0357
**GM-CSF**	7.4 ± 0.93531	9.81167 ± 2.057	9.75417 ± 0.63444	13.6125 ± 1.35175	0.0576
**IL-1β**	3.25833 ± 0.31944	3.09333 ± 0.4049	7.9375 ± 0.95513	11.2917 ± 1.19025	0.008
**IL-17F**	2.325 ± 0.08184	2.55167 ± 0.1537	5.36667 ± 0.28966	6.17083 ± 0.25629	0.0185

Supplemental Table 4. Viruses detected by PCR in oral swabs or fecal pellets of young (3-month-old) and old (20-24-month-old) female mice at day 7 post-exposure to NME. (MHV: mouse hepatitis virus; MNV: murine norovirus; TMEV: Theiler's encephalomyelitis virus; PV: parvovirus)

	MHV	MNV	TMEV	PV
**Young**	7/7	7/7	7/7	1/7
**Old**	7/7	7/7	7/7	0/7

Supplemental Table 5. Serological analysis of multiple viral infections in young (3-month-old) or old (21-22-month-old) female mice on day 21-23 that survived NME following prior immunization with a sublethal dose of MHV (see Fig. 3F). (MVM: minute virus of mice; MNV: murine norovirus; GDVII: TMEV: Theiler's encephalomyelitis virus; PV: parvovirus)

	MVM	MNV	TMEV	PV
**Young**	3/26	13/26	23/26	0/26
**Old**	1/24	12/24	6/24	1/24

Supplemental Table 6. Primers and probes for qRT-PCR

Human Taqman probes

TBP Hs00427620_m1

IL6 Hs00174131_m1

IL8 CXCL Hs00174103_m1

MCP-1 CCL2 Hs00234140_m1

IFITM1 Hs00705137_s1

IFITM2 Hs00829485_sH

IFITM3 Hs03057129_s1

ACE2 Hs1085330_m1

TMPRSS2 Hs01122322_m1

P16 Hs00923894_m1

P21 Hs00355782_m1

IL1a Hs00174092_m1

IL1b Hs01555410_m1

PAI1 Serpine1 Hs01126607_g1

IFN-a2 Hs00265051_s1

CSF2 Hs00929873-m1

CCL5 Hs00982282_m1

CXCR3 Hs01847760-s1

IL10 Hs00961622_m1

PAI2 Serpine2 Hs00299953_m1

Mouse Taqman probes

Cdkn1a Mm04205640_g1

Cdkn2a Mm00494449_m1

Gapdh Mm99999915_g1

Il1a Mm00439620_m1

Il6 Mm00446190_m1

Tnfa Mm00443258_m1

Mcp1 Mm00441242_m1

Mouse primers

Cdkn1a (*p21*^{Cip1})

Fwd 5'-GTCAGGCTGGTCTGCCTCCG-3'

Rev 5'-CGGTCCCGTGGACAGTGAGCAG-3'

Cdkn2a (*p16*^{Ink4a})

5'-CCCAACGCCCCGAACT-3'

Rev 5'-GCAGAAGAGCTGCTACGTGAA-3'

Cxcl1

Fwd 5'-ACCCGCTCGCTTCTCTGT-3'

Rev 5'-AAGGGAGCTTCAGGGTCAAG-3'

Cxcl2

Fwd 5'-CCTGGTTCAGAAAATCATCCA-3'

Rev 5'-CTTCCGTTGAGGGACAGC-3'

Cxcl10

Fwd 5'- GCCGTCATTTTCTGCCTCAT-3'

Rev 5'- GCTTCCCTATGGCCCTCATT-3'

Gapdh

Fwd 5'-AAGGTCATCCCAGAGCTGAA-3'

Rev 5'-CTGCTTCACCACCTTCTTGA-3'

Il1ß

Fwd 5'-CACAGCAGCACATCAACAAG-3'

Rev 5'-GTGCTCATGTCCTCATCCTG-3'

Il2

Fwd 5'-CCTGAGCAGGATGGAGAATTACA-3'

Rev 5'-TCCAGAACATGCCGCAGAG-3'

Il6

Fwd 5'-CTGGGAAATCGTGGAAT-3'

Rev 5'-CCAGTTTGGTAGCATCCATC-3'

Il7

Fwd 5’-TCTGCTGCCTGTCACATCATC-3’

Rev 5’-GGACATTGAATTCTTCACTGATATTCA-3’

Il10

Fwd 5’-ATAACTGCACCCACTTCCCA-3’

Rev 5’-GGGCATCACTTCTACCAGGT-3’

Il17

Fwd 5’-TCTCCACCGCAATGAAGACC-3’

Rev 5’-CACACCCACCAGCATCTTCT-3’

Mcp1

Fwd 5'-GCATCCACGTGTTGGCTCA-3'

Rev 5'-CTCCAGCCTACTCATTGGGATCA-3'

Mip1a

Fwd 5'-CCTTGCTGTTCTTCTCTGTACCATG-3'

Rev 5'-GCATTCAGTTCCAGGTCAGTGATG-3'

MHV

Fwd 5'-CAGATCCTTGATGATGGCGTAG-3'

Rev 5'-AGAGTGTCCTATCCCGACTTTCTC-3'

Pai1

Fwd 5'-GACACCCTCAGCATGTTCATC-3'

Rev 5'-AGGGTTGCACTAAACATGTCAG-3'

Pai2

Fwd 5'-ACTTAATGGGCTTTATCCTTTCC-3'

Rev 5'-TGCGTCCTCAATCTCATCG-3'

Tnfa

Fwd 5’-ATGAGAAGTTCCCAAATGGC-3’

Rev 5’-CTCCACTTGGTGGTTTGCTA-3’
